# Development of proteome-derived cyclic peptide libraries via ortho-phthalaldehyde-assisted cyclization

**DOI:** 10.1016/j.isci.2026.117598

**Published:** 2026-09-22

**Authors:** Ying Wei, Kangze Feng, Wenxuan Hou, Xiaolin Tian, Yuanyuan Xue, Wenhao Zhang, Haiteng Deng

**Affiliations:** 1 MOE Key Laboratory of Bioinformatics, Center for Synthetic and Systematic Biology, State Key Laboratory of Complex, Severe, and Rare Diseases, School of Life Sciences, Tsinghua University, Beijing, China; 2 Zhejiang Key Laboratory of Multiomics and Molecular Enzymology, Yangtze Delta Region Institute of Tsinghua University, Jiaxing 314006, China

**Keywords:** cyclic peptide library, ortho-phthalaldehyde, OPA, protein recognition, mass spectrometry, IgG-binding peptide

## Abstract

Cyclic peptides possess unique structural features and binding capabilities that make them attractive scaffolds for molecular recognition. However, strategies for generating functionally diverse libraries directly from natural proteomes remain comparatively underdeveloped. Here, we present a proteome-informed approach for constructing cyclic peptide libraries by integrating cysteine-enrichment protocols with ortho-phthalaldehyde (OPA)-mediated macrocyclization. This method enables direct library generation from proteome digests and is compatible with liquid chromatography-tandem mass spectrometry (LC-MS/MS)-based annotation and downstream functional screening. We systematically identify key parameters governing OPA-mediated cyclization topology and develop a rule-based pipeline for automated library annotation of OPA-cyclized peptides. Applying this strategy, we generate a library of over 15,000 unique cyclic peptides and perform target-specific screening against human IgG. This yielded cyclic peptide binder candidates, including one lead peptide showing cyclization-dependent IgG-binding potential. Our study establishes an OPA-enabled, proteome-informed workflow for cyclic peptide library construction and annotation.

## Highlights

• OPA cyclization builds cyclic peptide libraries from proteome digests
• Rule-based LC-MS/MS annotation assigns NT-loop and K-loop topologies
• Proteome-scale analysis identifies 15,217 unique OPA-generated cyclic peptides
• Affinity screening identifies a cyclization-dependent IgG-binding candidate

## Teaser

Biochemistry; Biochemistry methods; Proteomics

## Introduction

Cyclic peptides represent a structurally constrained class of molecules with broad applications in chemical biology, diagnostics, and drug discovery.^1^^,^^2^^,^^3^^,^^4^ Their rigid conformations often confer improved binding affinity, stability against proteolysis, and selective recognition of protein surfaces compared to their linear counterparts. Consequently, cyclic peptides have been investigated as therapeutic leads, molecular probes, and antibody mimetics.^5^^,^^6^ Realizing the full potential of cyclic peptides, however, requires effective strategies for generating diverse libraries together with robust methods for screening and identification.

Current approaches to cyclic peptide library construction include solid-phase peptide synthesis (SPPS),^7^ enzymatic or ribosomal cyclization,^8^ and display technologies, such as phage or mRNA display.^9^^,^^10^^,^^11^^,^^12^^,^^13^^,^^14^ These platforms have enabled major advances in cyclic peptide discovery, and recent methodological developments have substantially expanded both library scale and downstream decoding capability. For example, fast-flow synthesis has enabled access to highly diverse synthetic libraries spanning chemical spaces at the 10^∧^18 scale, with synthesis times reduced to less than 1 h.^15^ In parallel, linearizable or chemically linearizable macrocyclic peptide formats have improved post-selection mass spectrometry (MS) readability and *de novo* sequencing of macrocyclic peptide mixtures.^16^^,^^17^^,^^18^^,^^19^^,^^20^ Related affinity-selection MS workflows have also used reversibly reactive peptide formats to enrich and identify covalent peptide binders directly from synthetic libraries.^21^ Nevertheless, these strategies differ in their dependence on predefined synthetic library formats, encoded sequence space, cyclization chemistry, and post-selection decoding workflow. In particular, complementary approaches that directly access native proteolytic sequence space while remaining compatible with liquid chromatography-tandem mass spectrometry (LC-MS/MS)-based annotation remain of considerable interest.

Ortho-phthalaldehyde (OPA) chemistry offers a potentially useful route in this context. OPA reacts rapidly and selectively with thiols and primary amines to form stable isoindole derivatives under mild conditions.^22^^,^^23^ This orthogonal reactivity suggests that cysteine-containing peptides may be cyclized through OPA-mediated condensation, without the need for protecting groups or harsh reagents.^24^ Importantly, OPA cyclization is compatible with aqueous environments and MS analysis, making it well suited for proteome-derived substrates. Yet, despite the long-standing use of OPA in amino acid (aa) labeling and protein derivatization, its potential for constructing large-scale cyclic peptide libraries has not been systematically explored.

Here, we present a proteome-informed strategy for generating cyclic peptide libraries via OPA-mediated macrocyclization. Our workflow begins with selective enrichment of cysteine-containing peptides from complex proteome digests, followed by OPA-mediated ring closure under mild aqueous conditions. The resulting libraries, comprising more than 15,000 unique members, were characterized by LC-MS/MS together with a rule-based fragmentation framework that supports automated annotation of OPA-cyclized peptides. As a proof of concept, we applied this workflow to human immunoglobulin G (IgG)^25^^,^^26^^,^^27^^,^^28^ and identified cyclic peptide-binder candidates using surface plasmon resonance (SPR),^29^ hydrogen-deuterium exchange mass spectrometry (HDX-MS),^30^^,^^31^ molecular docking, and serum pull-down assays.

Rather than replacing existing cyclic peptide library platforms, this study introduces a complementary workflow that links chemical macrocyclization with proteomic-scale peptide discovery. By integrating cysteine enrichment, OPA-mediated chemistry, and LC-MS/MS-based analysis, we expand the available toolkit for cyclic peptide research and provide a proteome-compatible route for identifying protein-binding peptide candidates from native sequence space. An overview of the proteome-derived cyclic peptide library construction and targeted ligand discovery workflow is illustrated in Scheme 1.

**Scheme 1:**
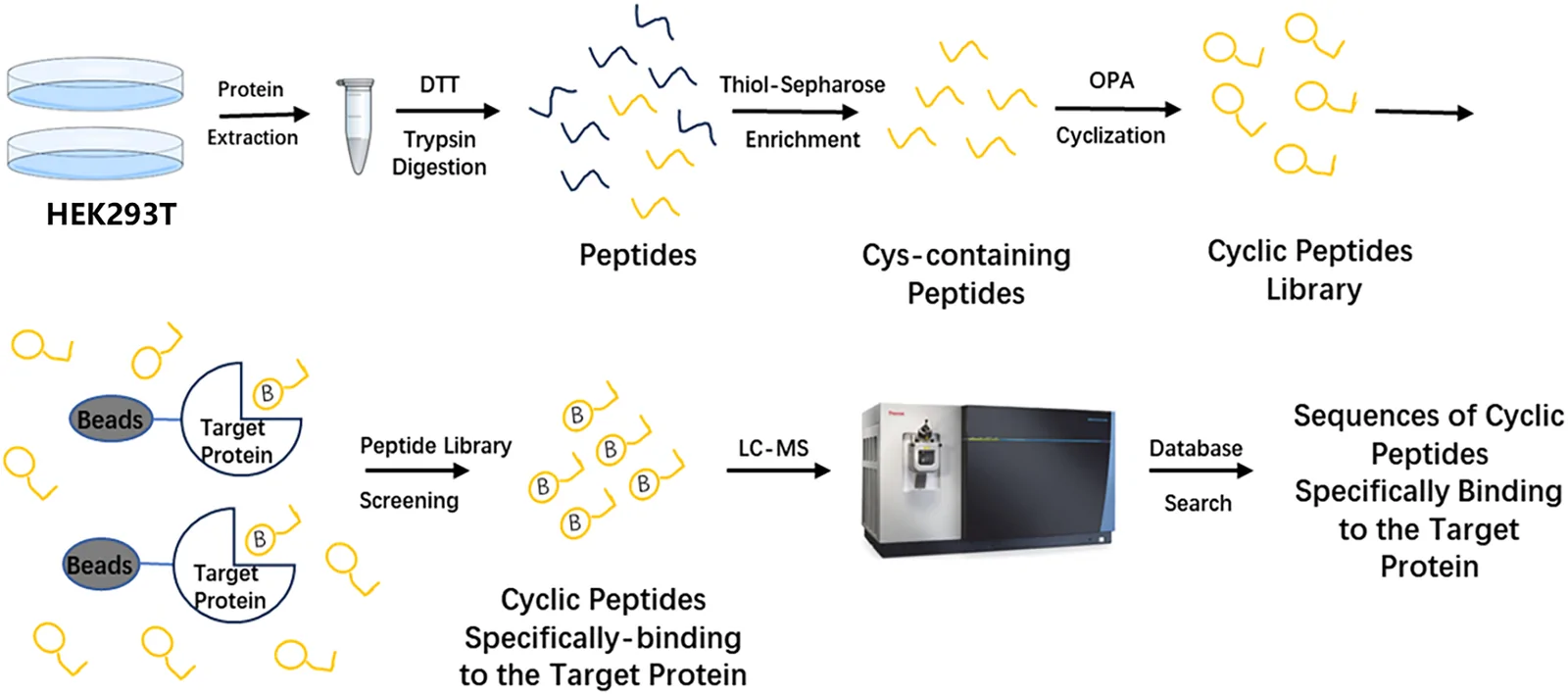
Workflow for proteome-derived cyclic peptide library construction and target protein binder candidate identification

## Results

### Selective enrichment of cysteine-containing peptides

Proteome digests from HEK-293T cells initially yielded 20,539 identified peptides, of which 655 (3.19%) were Cys-containing peptides detected in the unenriched global digest under standard LC-MS/MS sampling depth. Following enrichment using pyridyl disulfide-activated thiopropyl resin, the purity of Cys-containing peptides increased to 86.9%, corresponding to a >25-fold enrichment (Figures 1A and 1B). Analysis of the enriched fraction yielded 3,326 unique Cys-containing peptides in total, indicating substantially improved detectability of low-abundance Cys peptides after enrichment and providing a focused substrate pool for subsequent OPA cyclization (Table S1). Notably, the composition of the enriched peptide set was broadly consistent with the inherent reactivity profile of OPA. Kinetic profiling showed that Cys was the most reactive nucleophilic residue toward OPA (>95% conversion within 10 min), whereas acidic residues such as Asp and Glu exhibited negligible reactivity (Figure S8). Compositional analysis (Figure 1C) further showed that the enrichment procedure increased the proportion of highly OPA-reactive Cys-containing peptides while reducing the relative abundance of low-reactivity acidic residues. Together, these observations suggest that the enrichment step produces a peptide pool that is likely to be favorable for subsequent OPA-mediated macrocyclization.

**Figure 1:**
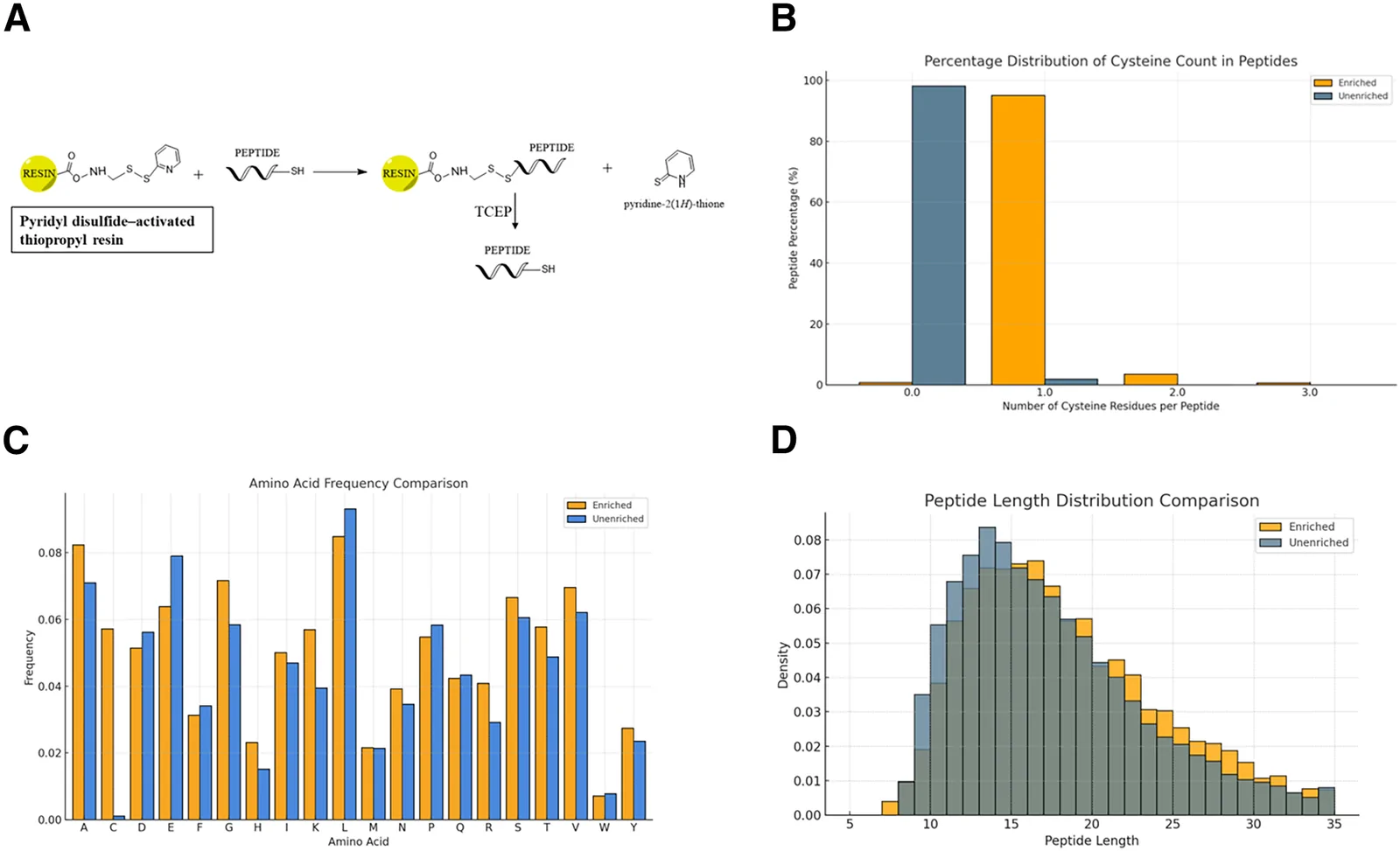
Selective enrichment of cysteine-containing peptides (A) Workflow of thiol-affinity enrichment using pyridyl disulfide-activated thiopropyl resin. Thiol groups react with activated disulfides on the resin surface, enabling enrichment prior to OPA cyclization. (B) Amino acid composition of peptides before (blue) and after (yellow) cysteine enrichment. Cysteine enrichment resulted in a marked increase in cysteine-containing peptides. In the enriched group, the majority of peptides contained a single cysteine residue, with fewer peptides containing two or three cysteine residues. (C) Amino acid frequency comparison between enriched and unenriched peptide pools, showing an increased proportion of cysteine residues and a reduced abundance of acidic residues (D and E) in the enriched sample. (D) Comparative analysis of peptide length distribution. The enriched pool exhibits a characteristic unimodal distribution centered at 14–16 residues. Statistical significance was determined by the Kolmogorov-Smirnov (KS) test (KS statistic = 0.070, *p* = 1.66 × 10^−12^), indicating a significant enrichment of longer peptides (10–20 aa) compared to the initial proteome digest.

We next evaluated the physical characteristics of the enriched peptides. Statistical analysis revealed a significant shift toward longer sequences following enrichment, with the majority of peptides distributed between 10–20 aa (Figure 1D). This length distribution is intrinsically advantageous for our cyclic library; peptides within this range not only facilitate robust LC-MS/MS identification but also provide the conformational flexibility required to accommodate the steric constraints of OPA-mediated ring closure. Despite the observed bias toward medium-to-long fragments, the library maintained structural diversity, spanning 6–35 residues.

### OPA-mediated cyclization generates identifiable NT-loop and K-loop products under mild aqueous conditions

To establish an analytical framework for identifying OPA-cyclized peptides, we first examined the fragmentation chemistry of OPA-mediated condensation using a model peptide (AAAAACAAAAK). This reaction yielded two distinct macrocyclic topologies: NT-loop (via N-terminal α-amine) and K-loop (via lysine ε-amine) (Figure 2A). Interrogation via high-resolution MS/MS revealed abundant and well-distributed b- and y-type ions, including diagnostic α-cleavages flanking the OPA linker (Figures 2B and 2C). We found that the cleavage of the C–S bond during MS/MS scanning generated characteristic mass offsets, including a +130.9950 Da modification on amine-bearing fragments and a corresponding −32.9793 Da loss on the cysteine residue, consistent with dehydroalanine formation (Figures 3A and 3B). Based on these fragmentation features, peptide topologies could be assigned within the analytical framework used in this study. NT-loop configurations were supported by the persistent +130.9950 Da offset across the b-ion series, whereas K-loop topologies were assigned when this offset appeared on internal or y-type fragments containing the modified lysine.

**Figure 2:**
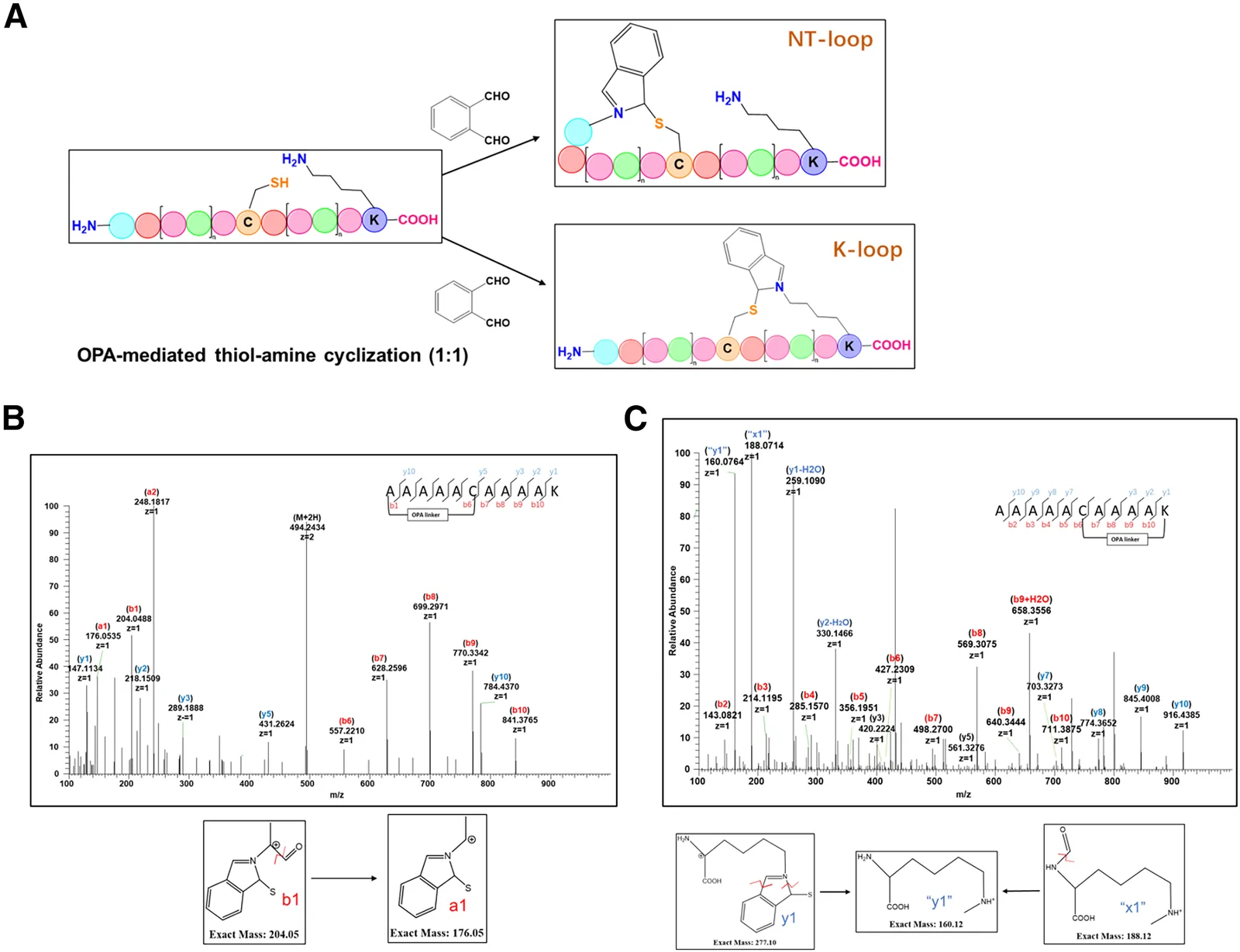
OPA-mediated cyclization and fragmentation of model peptide (A) Reaction scheme of OPA condensation between cysteine thiol and amino groups. Two possible cyclization topologies observed in the model peptide: NT-loop (N-terminal α-amine) and K-loop (lysine ε-amine). (B) Representative MS/MS spectrum of an OPA-cyclized model peptide (NT-loop). The HCD spectrum is annotated with b- and a-ions (red) and y-ions (blue), with the doubly charged precursor at (M+2H)^2+^ = 494.2434, and the OPA linker indicated. The fragmentation pattern reveals the OPA-modified thioisoindole ring, showing the formation of diagnostic b1 and a1 ions, which support the NT-loop assignment. (C) Representative MS/MS spectrum of an OPA-cyclized model peptide (K-loop). The HCD spectrum is annotated with b-ions (red) and y- and x-ions (blue). The fragmentation pattern reveals the formation of a diagnostic y1 ion; high-energy collisions induced isoindole ring opening, generating characteristic y1 and x1 ions, which support the K-loop assignment.

**Figure 3:**
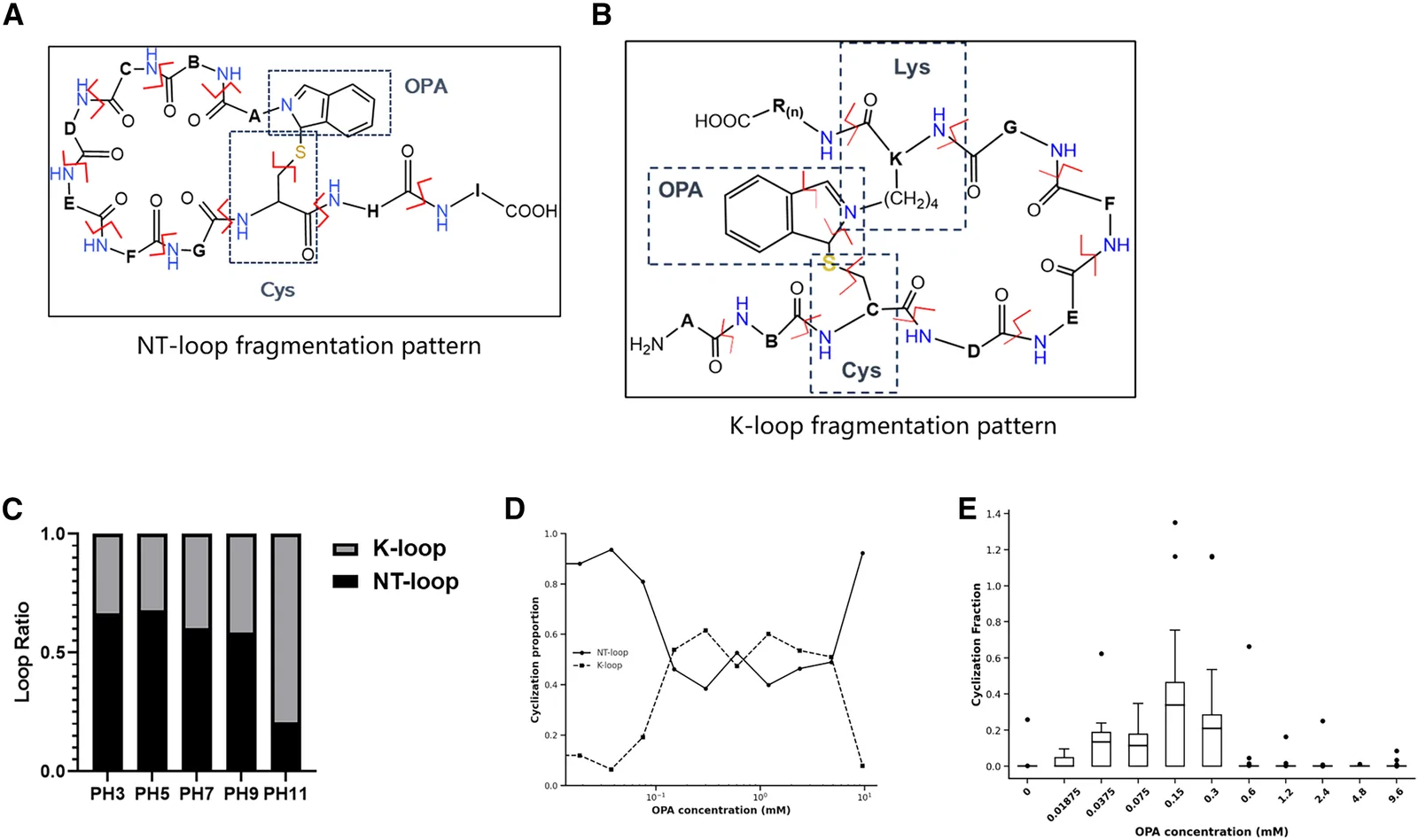
Fragmentation rules and reaction parameter control of OPA-mediated peptide cyclization (A and B) OPA-mediated NT-loop and K-loop fragmentation patterns. Schematic representation of two typical cyclic structures formed by OPA condensation with cysteine thiol and either the N-terminal α-amine (A) or lysine ε-amine (B). Model sequences (ABCDEFGCHI and ABCDEFGKR) are shown as examples for illustrating topology and do not correspond to actual peptides. Red dashed lines indicate preferential cleavage sites under CID/HCD conditions. In both cases, fragmentation occurs at the Cys αC–S bond adjacent to the OPA linker, but the resulting ions exhibit topology-specific mass shifts. This schematic highlights diagnostic differences in fragmentation patterns between NT-loop and K-loop configurations, providing a theoretical basis for structural annotation in MS/MS analysis. (C) Effect of pH on loop topology distribution in OPA-mediated cyclization: Distribution of NT-loop and K-loop topologies across a pH gradient. NT-loop formation is favored under mildly acidic to neutral conditions, including pH 7.4, whereas K-loops increase under alkaline conditions. (D) Control of NT-loop and K-loop ratios by OPA concentration: Topology distribution as a function of OPA concentration. Low OPA favors NT-loop (>80%), while 0.15 mM shifts dominance to K-loop (∼62%). At high OPA (≥0.6 mM), both topologies balance (∼40%–60%). (E) Effect of OPA concentration on cyclization extent in BSA-derived peptide libraries: extent of cyclization in BSA-derived peptide libraries under varying OPA concentrations. The highest conversion under the tested concentrations was observed at 0.15–0.30 mM, with reduced conversion at higher concentrations.

Having established the fragmentation rules of OPA-cyclized peptides, we next assessed how reaction conditions influence cyclization outcomes and evaluated these principles in a natural proteolytic context. For the model peptide AAAAACAAAAK, pH had a clear effect on loop topology preference: NT-loops predominated under acidic-to-neutral conditions (pH 5–7), including at physiological pH 7.4, whereas K-loop products became more frequent under alkaline conditions (pH ≥ 9) (Figure 3C).

To extend these observations to a more complex system, bovine serum albumin (BSA) was selected as a model protein because of its well-defined sequence, predictable digestion pattern, commercial availability, and enrichment in cysteine and lysine residues. Digestion of BSA generated a representative peptide library containing typical modification sites, thereby mimicking the diversity of natural proteomic substrates. By incorporating the fragmentation rules described previously into the search workflow (pLink 3^32^), 24 OPA-cyclized peptides were identified from these small libraries (Figure S1; Table S2). In this system, OPA concentration had a pronounced effect on the extent of cyclization, showing minimal conversion at ≤0.05 mM, maximal conversion at 0.15–0.30 mM, and reduced conversion at higher concentrations (≥0.6 mM) (Figure 3E). For peptides capable of forming both NT- and K-loops, a concentration-dependent shift was also observed: NT-loops dominated at very low OPA concentrations, K-loops peaked at ∼0.15 mM, and both topologies approached parity at intermediate-to-high concentrations. At the highest concentration tested (9.6 mM), NT-loops again became predominant (Figure 3D). Taken together, these results suggest that both pH and OPA concentration influence loop topology outcomes, with near-neutral pH and an OPA concentration of ∼0.15 mM providing favorable conditions for library construction under the tested system.

### Construction and characteristics of the natural OPA-derived cyclic peptide library

Leveraging the optimized reaction conditions and automated annotation workflow established previously, we next constructed a natural OPA-derived cyclic peptide library from the HEK-293T proteome. Based on *in silico* enumeration, approximately 7.7% of tryptic peptides (95,277 sequences) are theoretically cyclizable. Following thiol-affinity enrichment and OPA-mediated cyclization, we identified 15,217 unique cyclic peptides (FDR <5%, Table S3), including 9,053 NT-loop and 6,164 K-loop configurations (Figure 4A). OPA-mediated condensation can also give rise to intermolecular crosslinking and peptide oligomerization, particularly at elevated reagent concentrations or peptide densities (Table S3). In the present study, downstream analysis was focused on confidently identified intramolecular macrocycles, whereas intermolecular products are described separately in the supplemental information.

**Figure 4:**
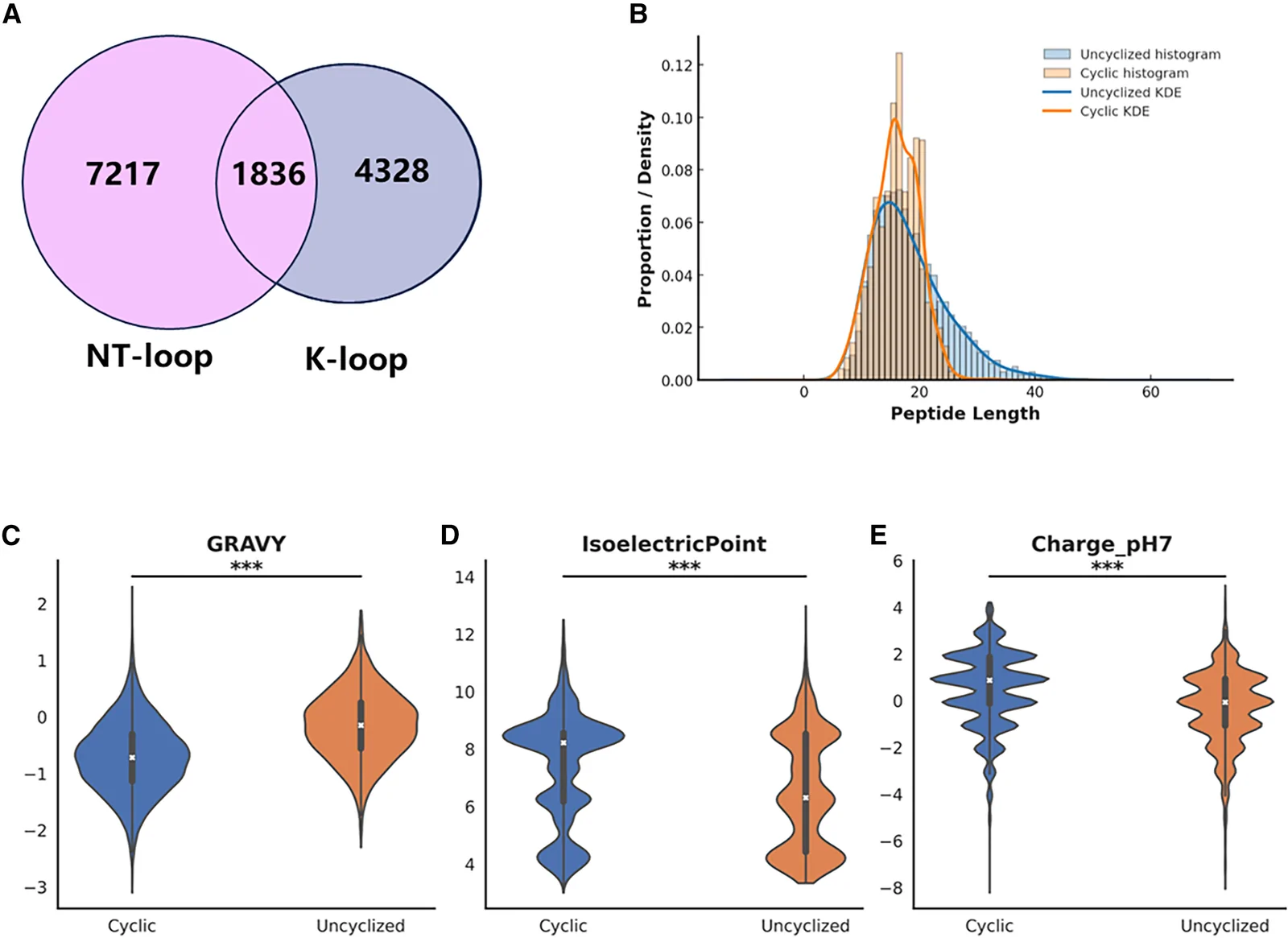
Properties of the OPA-cyclized peptide library (A) Distribution of NT-loop and K-loop topologies in the OPA-cyclized peptide library, comprising 15,217 unique cyclic peptides identified from proteome digests after OPA cyclization. (B) Length distribution of uncyclized and cyclic peptides. Histogram and kernel density estimation (KDE) curves comparing peptide lengths in uncyclized (blue) and OPA-cyclized (yellow) groups. Uncyclized peptides showed a broader distribution, including both short (∼6–10 aa) and longer (∼14–18 aa) species, with a tail extending to higher lengths. In contrast, cyclic peptides were more concentrated, with a dominant peak at ∼9–11 aa and markedly fewer long peptides, consistent with preferential recovery of intermediate-length cyclic peptides. (C–E) Physicochemical distribution of GRAVY, isoelectric point (pI), and net charge at pH 7 in uncyclized and cyclic groups. The cyclic group (cyclic) exhibits an average GRAVY value of −0.71 (SD = 0.63), whereas the uncyclized group (uncyclized) shows an average of −0.15 (SD = 0.60). The cyclic group (cyclic) has an average pI of 7.36 (SD = 1.89), while the uncyclized group (uncyclized) has an average pI of 6.48 (SD = 1.97). The cyclic group (cyclic) shows an average net charge of 0.49 (SD = 1.60), compared to −0.35 (SD = 1.56) in the uncyclized group (uncyclized). Each violin plot represents the distribution of the corresponding parameter in the peptide library, with the nested boxplot displaying the median and interquartile range (IQR). Statistical significance was assessed using a two-tailed Welch’s *t* test (∗*p* < 0.05, ∗∗*p* < 0.01, ∗∗∗*p* < 0.001). The outer contour of each violin plot represents the kernel density estimate, with the width corresponding to the relative frequency within each value range.

Analysis of aa composition showed that OPA-reactive residues, particularly Cys, Trp, and His, were enriched in the cyclic group relative to the uncyclized group (Figure S2), with Cys showing the most pronounced enrichment. This trend was also reflected in the Cys residue count distribution, in which the cyclic library contained a higher proportion of peptides with two or more Cys residues (Figure S2). These observations are consistent with the high intrinsic reactivity of Cys toward OPA and with the increased probability of successful ring closure provided by multiple thiol sites.

Peptide length distribution, visualized via kernel density estimation (KDE), showed that cyclic peptides were predominantly concentrated within the 9–12 aa range (peak at 9–11 aa), whereas uncyclized peptides exhibited a broader profile (Figure 4B). This apparent “sweet spot” at intermediate peptide length may reflect the spatial constraints of OPA-mediated cyclization, where longer peptides potentially face higher entropic costs for ring closure, whereas shorter sequences are likely to form more stable structures. Further analysis of the “loop span” indicated a preference for compact architectures: over 75% of NT-loops closed within 2–10 aa, while K-loops exhibited an even stronger bias toward proximal sites (85% within 1–7 aa), suggesting that K-loop formation may be more sensitive to spatial proximity (Figure S3).

Physicochemical profiling further showed that OPA-cyclized peptides exhibited a centralized distribution compared to uncyclized counterparts, characterized by higher hydrophilicity (mean GRAVY = −0.71 vs. −0.15) and narrower ranges for both isoelectric point (pI) (6.5–8.5) and net charge (−1 to +2) (Figures 4C–4E). This convergence toward neutral-to-slightly alkaline and hydrophilic properties may be compatible with favorable solvation of the isoindole-based bridge and may also contribute to the observed analytical behavior of the cyclic peptide set. Evaluation of MS/MS performance through Alpha fragment matching and sequence coverage further demonstrated the annotation reliability of the dataset; notably, the K-loop configuration outperformed the NT-loop (*p* < 0.001), suggesting more uniform diagnostic fragment distribution and superior identification sensitivity under the current workflow (Figure S4). Furthermore, Gene Ontology (GO) enrichment analysis indicated a shift in protein origins from cytoskeletal and contractile proteins in the uncyclized group toward extracellular matrix (ECM) and basement membrane components in the cyclic library (Figure S5). This bias may reflect the higher cysteine density and reduced conformational entropy of ECM-derived peptides, which could favor macrocyclization. Collectively, these results indicate that the OPA-derived library captures chemically and biologically distinct features within the accessible proteomic sequence space.

### Affinity screening against human IgG identifies cyclic peptide binder candidates

Affinity selection of the OPA-derived cyclic peptide library against immobilized human IgG yielded multiple high-confidence candidate binders. Among these candidates (Table 1), IgG-P1-loop (QPGALTQGHSCSR), derived from a putative G-protein-coupled receptor (GPR32P1), was prioritized for downstream validation because of its assigned NT-loop topology, favorable hydrophilicity (GRAVY = −0.869), and MS/MS identification score. The annotated HCD MS/MS spectrum of IgG-P1-loop showed a diagnostic a1 ion supporting the assigned OPA-linked NT-loop topology, with a precursor mass error of −2.25 ppm (Figure 5C).

**Table 1:** Potential IgG binding candidate cyclic peptides identified by pLink 3

Potential binding peptide ID	Cyclization type and position	Length	Cys count	GRAVY	Score	Precursor ion mass error (ppm)
IgG-P1-loop	NT-loop (1–11)	13	1	−0.869	0.0219	−2.25
IgG-P2-loop	NT-loop (1–10)	20	2	−0.855	0.0136	−2.16
IgG-P3-loop	NT-loop (1–15)	29	2	0.631	0.0031	6.14

IgG-P1-loop sequence: QPGALTQGHSCSR, derived from the putative G-protein-coupled receptor GPR32P1. IgG-P2-loop sequence: ESPNFESYTCLCAPGWQGQR, derived from the Notch signaling pathway protein Notch2. IgG-P3-loop sequence: IDAASPLEKVCLIGCGFSTGYGSAVNVAK, derived from alcohol dehydrogenase ADH1A or ADH1B. No known reports of direct IgG binding for any of the peptides’ protein sources. After a comprehensive comparison of the sequence characteristics and mass spectrometry parameters, IgG-P1-loop (QPGALTQGHSCSR) was selected as the focus for further validation.

**Figure 5:**
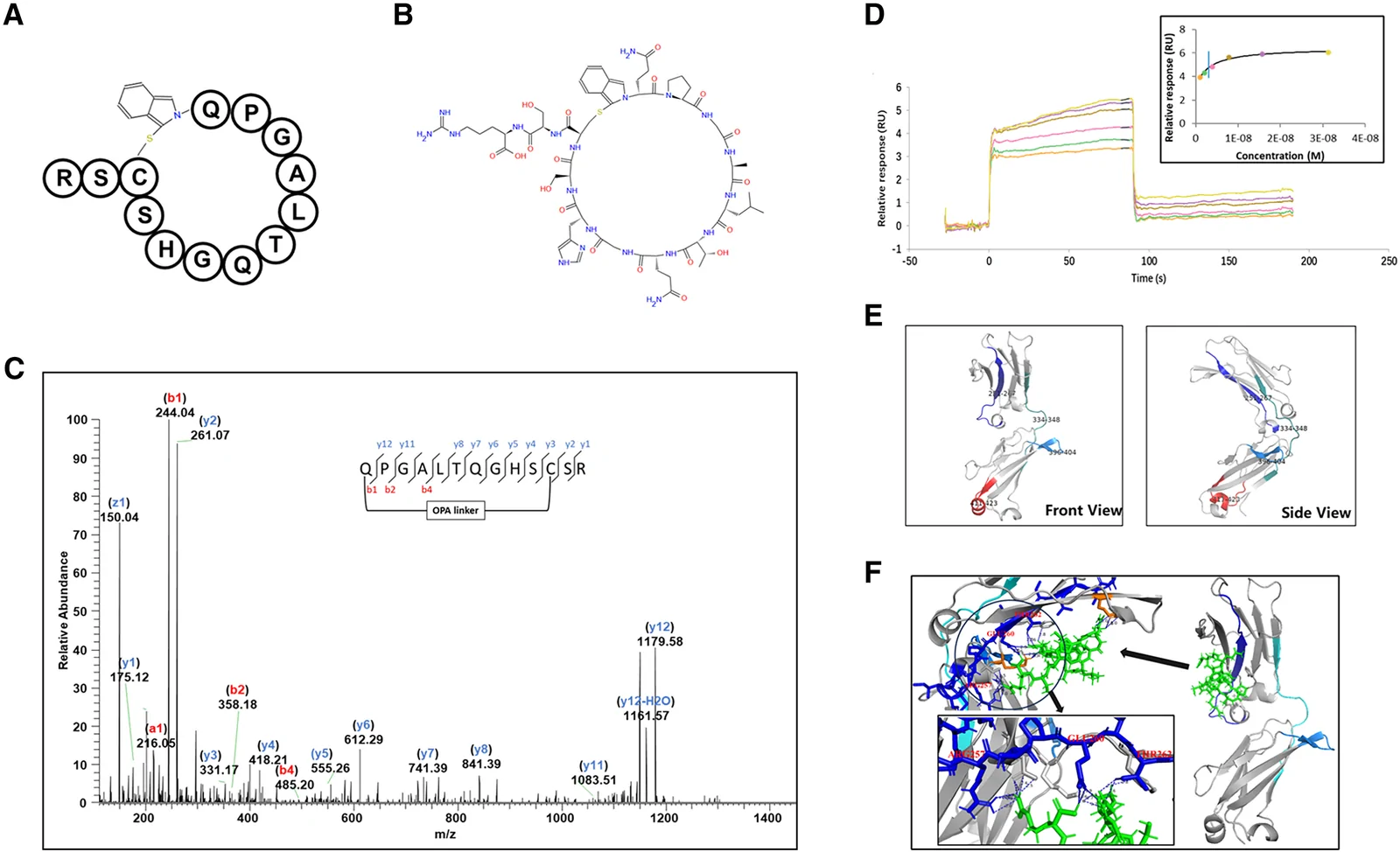
Structural characterization and binding-related analysis of IgG-P1-loop (A) Schematic representation of the assigned NT-loop topology of IgG-P1-loop. The OPA-derived linker connects the N-terminal amine and the cysteine thiol group. (B) Chemical structure of IgG-P1-loop. (C) Annotated HCD MS/MS spectrum of IgG-P1-loop obtained from the affinity-selection eluate by high-resolution LC-MS/MS analysis. Matched a- and b-type fragment ions are labeled in red, y-type fragment ions are labeled in blue, and green markers indicate the positions of matched fragment-ion peaks. The diagnostic a1 ion supports the assigned OPA-linked NT-loop topology. The OPA-mediated cyclization site is indicated as “OPA linker” in the sequence schematic. The precursor mass error was −2.25 ppm. (D) Surface plasmon resonance (SPR) sensorgrams of IgG-P1-loop measured against immobilized human IgG at the indicated peptide concentrations. The inset shows the corresponding steady-state affinity fitting, with the fitted curve shown as a black solid line. Steady-state affinity fitting yielded an apparent SPR-determined dissociation constant (*K*_D_) of 3.12 nM, with an *R*_max_ value of 3.3 RU. (E) Hydrogen-deuterium exchange mass spectrometry (HDX-MS) mapping of differential deuterium uptake onto the Fc region of human IgG (PDB: 5W5L). Blue and teal segments indicate regions with decreased deuterium exchange, including residues 253–269, 336–350, and 398–406, whereas red segments indicate regions with increased exchange, including residues 413–425. Residues 57–60 are not shown because the corresponding coordinates are unresolved in the structure. (F) DockThor-predicted model of the IgG Fc-IgG-P1-loop complex. IgG-P1-loop is shown in green, the IgG backbone is shown in gray, and HDX-protected regions are highlighted in blue and teal. The model positions IgG-P1-loop near the 253–269 region, consistent with the HDX–MS protection pattern. The enlarged view highlights predicted contacts involving Arg257, Glu260, and Thr262. Dashed lines indicate predicted hydrogen bonds, with donor–acceptor distances labeled in Å.

To further evaluate this candidate, the linear precursor peptide of IgG-P1-loop, IgG-P1-Line, was obtained via commercial solid-phase peptide synthesis, followed by OPA-mediated cyclization in PBS buffer (pH 7.4) to generate IgG-P1-loop with suitable purity for downstream binding experiments (Figure S9). SPR analysis showed concentration-dependent binding responses of IgG-P1-loop toward immobilized human IgG under the tested assay conditions. Steady-state affinity fitting yielded an apparent SPR-determined *K*_D_ of 3.12 nM (Figure 5D). Similar binding trends were also observed for rabbit, rat, and mouse IgG in the same assay format (Figures S6A–S6C). This cross-species reactivity may be consistent with recognition of a relatively conserved region within the IgG constant domain. By contrast, the linear precursor IgG-P1-Line produced substantially weaker responses toward human IgG under the same assay conditions, and its concentration-dependent data did not support reliable steady-state affinity fitting (Figure S6D). This difference is consistent with the possibility that OPA-mediated macrocyclization stabilizes a binding-competent conformation. However, given the low signal intensity observed in all conditions and the quasi-instantaneous association and dissociation events, coupled with the absence of a conclusive negative control, we are unable to assess or determine target selectivity based on the presented SPR data.

To investigate the binding interface, HDX-MS was employed, achieving 94% sequence coverage of the IgG heavy chain (Figure S7A). IgG-P1-loop binding was associated with decreased deuterium exchange within the CH2 and CH3 domains, particularly in regions 253–269 (LMISRTPEVTCVVVDVS), 336–350, and 398–406 (Table 2; Figure 5E). These protection patterns, especially near the CH2–CH3 junction, are consistent with previously recognized Fc-binding regions. Conversely, localized increases in exchange at residues 413–425 and 57–60 may reflect compensatory conformational flexibility or allosteric rearrangements upon peptide binding. These observations were further supported by computational docking, which positioned IgG-P1-loop within a shallow groove on the Fc surface. The model identified a network of stabilizing interactions, including five hydrogen bonds (Table 3). Specifically, Arg257, Glu260, and Thr262 (Figure 5F)—all situated within the 253–269 HDX-protected region—were predicted to form multiple contacts with the peptide, providing a possible structural rationale for the interaction observed by SPR. Taken together, the HDX-MS protection pattern and docking model suggest that the reduced binding signal of the linear precursor may arise from its greater conformational flexibility. Without the constraint introduced by OPA-mediated cyclization, IgG-P1-Line may be less able to maintain a binding-competent presentation of its interacting residues and to engage the predicted Fc-surface interaction network. This interpretation remains a structural hypothesis, as the contributions of individual residues and the conformational ensemble of the linear precursor were not directly examined. Collectively, these results support the view that OPA-mediated cyclization can yield protein-binding candidates from proteome-derived sequences and that IgG-P1-loop represents a cyclization-dependent IgG-binding candidate under the present workflow.

**Table 2:** ΔHDX changes of the five most significant peptides in the binding of human IgG and IgG-P1-loop

Position	Sequence	ΔHDX (30s)	ΔHDX (90s)	ΔHDX (300s)	ΔHDX (600s)	Mean |ΔHDX|
253–269	LMISRTPEVTCVVVDVS	−0.64	−1.32	−2.04	−2.07	1.52
398–406	PVLDSDGSF	−0.61	−1.05	−0.34	−0.54	0.64
57–60	NKHY	+0.51	+0.41	+0.45	+0.50	0.47
413–425	TVDKSRWQQGNVF	+0.98	+0.21	+0.32	+0.25	0.44
336–350	KTISKAKGQPREPQV	+0.04	−0.58	−0.56	−0.35	0.38

Peptides were ranked by the mean absolute ΔHDX values across the four time points. The peptide 253–269 (LMISRTPEVTCVVVDVS) showed the largest ΔHDX reduction, consistent with a candidate binding region for IgG-P1-loop. Peptides such as 398–406 (PVLDSDGSF) showed similar protection patterns, whereas peptides 413–425 and 57–60 showed increased ΔHDX, which may reflect increased flexibility or conformational adjustment upon binding.

**Table 3:** Hydrogen bond interactions between IgG and IgG-P1-loop identified by PLIP

Residue site	Interaction type	Hydrogen bond distance (Å)	Donor angle (°)	Description
Lys248	hydrogen bond	2.18	103.68	backbone donor
Arg257	hydrogen bond	2.55	109.72	side-chain donor
Glu260	hydrogen bond + salt bridge	1.63	126.27	backbone receptor
Thr262	hydrogen bond	2.26	145.08	threonine O-H donor
Arg303	hydrogen bond	2.78	103.05	backbone donor

Key residues Arg257, Glu260, and Thr262 are located within the HDX-MS-protected region (253–269) and are included in the proposed ligand-binding interface. In the docking model, Arg257 is predicted to form a donor-acceptor hydrogen bond, Glu260 is predicted to participate in hydrogen bonding and salt bridge interactions, and Thr262 is predicted to form hydrogen-bonding contacts that may contribute to stabilization of peptide binding.

### Biochemical evaluation and cellular labeling performance of IgG-P1-loop

The practical utility of IgG-P1-loop was further examined through biochemical enrichment and cellular imaging assays. In pull-down experiments using complex human serum, resin-immobilized IgG-P1-loop enriched endogenous IgG heavy chains (∼55 kDa), whereas the linear precursor peptide (IgG-P1-Line) showed substantially reduced binding signal (Figures 6A–6C). These results are consistent with the idea that OPA-mediated macrocyclization may stabilize a binding-competent conformation and improve the spatial presentation of residues involved in IgG recognition.

**Figure 6:**
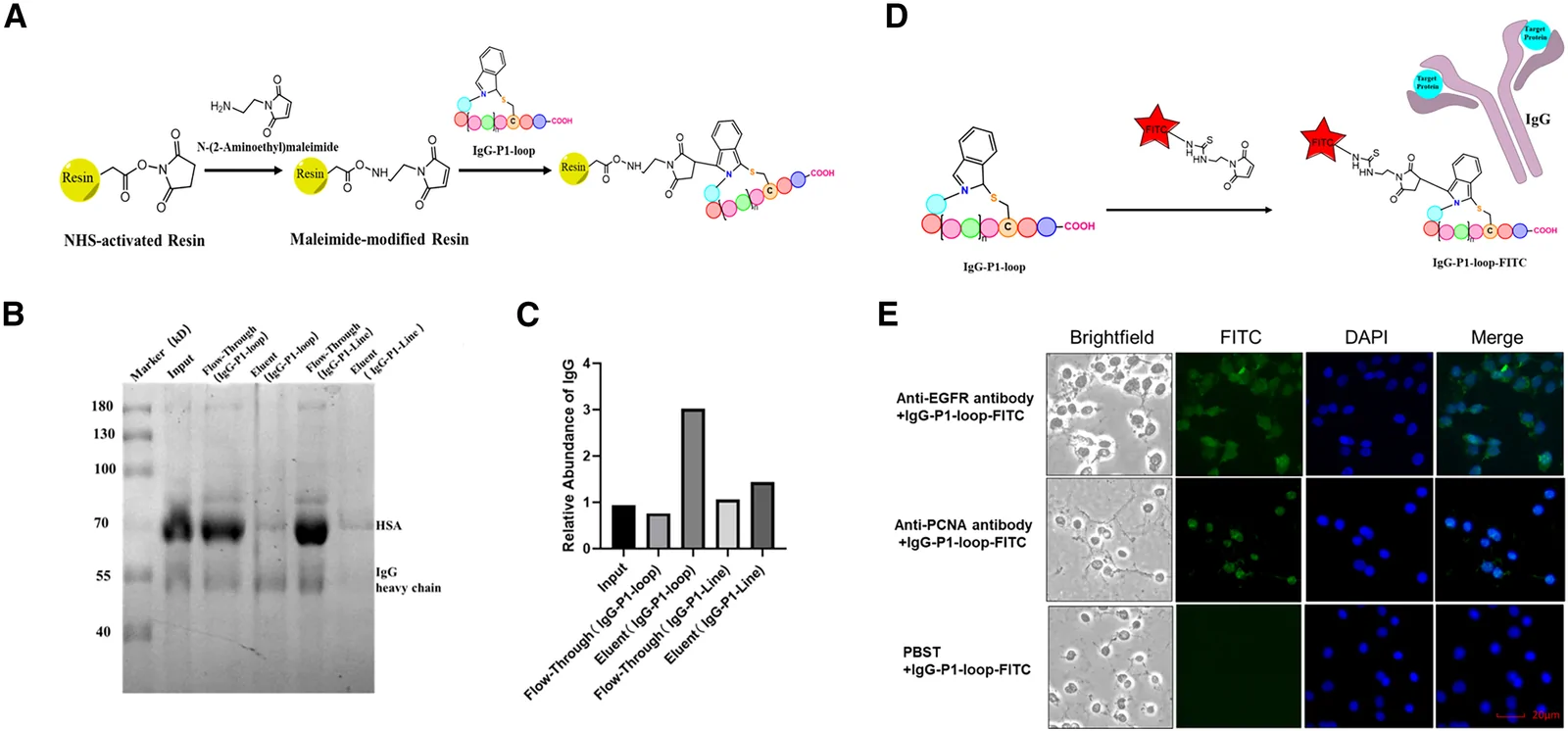
Biochemical evaluation and cellular labeling performance of IgG-P1-loop (A) Schematic of resin coupling: NHS-activated resin was modified with N-(2-aminoethyl)maleimide to yield maleimide-functionalized resin, which was subsequently conjugated to OPA-cyclized IgG-P1-loop to generate peptide-immobilized resin. (B) Pull-down of endogenous IgG from human serum using IgG-P1-loop resin versus linear control (IgG-P1-Line). SDS-PAGE of eluates shows a prominent IgG heavy-chain band at ∼55 kDa in the cyclic peptide group, but only background levels with the linear control. (C) Densitometric quantification of IgG heavy-chain bands normalized to human serum albumin (HSA) confirms significantly higher IgG enrichment by IgG-P1-loop compared to the linear control. (D) Schematic of probe generation: IgG-P1-loop was conjugated to fluorescein maleimide (FITC) to yield IgG-P1-loop-FITC, for fluorescence detection of antibody-dependent labeling. (E) Fluorescence imaging of HNEpCs treated with anti-PCNA or anti-EGFR primary antibodies followed by IgG-P1-loop-FITC (green) labeling. Antibody-dependent fluorescence signals were observed in the presence of primary antibodies, whereas antibody-free controls showed minimal background. DAPI (blue) counterstaining marks nuclei in all groups. Scale bars: 20 μm.

Beyond biochemical enrichment, we explored the feasibility of IgG-P1-loop as a secondary-antibody-like labeling probe. When conjugated with fluorescein (5-FITC-maleimide), the resulting IgG-P1-loop-FITC probe produced antibody-dependent fluorescence labeling in human nasal epithelial (HNEpCs) cells (Figure 6D). In cells pre-incubated with anti-PCNA or anti-EGFR primary antibodies, fluorescence signals were observed in patterns consistent with target-associated labeling, whereas controls lacking primary antibodies showed minimal background signal (Figure 6E). Together, these observations support the potential utility of IgG-P1-loop in IgG-dependent biochemical enrichment and labeling applications under the current experimental framework.

## Discussion

Cyclic peptides are privileged scaffolds in molecular recognition and drug development due to their rigid conformations, enhanced proteolytic stability, and high-binding specificity.^1^^,^^33^ Despite these advantages, strategies for generating structurally diverse cyclic peptide libraries directly from natural proteolytic sources remain comparatively limited. Here, we introduce a proteome-informed platform that integrates cysteine peptide enrichment with OPA-mediated condensation to generate chemically diverse, MS-compatible cyclic peptide libraries.

Unlike fully synthetic or genetically encoded platforms,^6^^,^^33^^,^^34^^,^^35^ the present workflow operates directly on proteolytic digests and therefore accesses endogenous peptide sequence diversity without requiring prior sequence encoding. Whereas synthetic approaches can access substantially larger predefined chemical spaces, the present workflow yielded 15,217 unique OPA-cyclized peptides from proteome digests with LC-MS/MS-based annotation. These strategies therefore address distinct but complementary experimental needs. The dual reactivity of OPA toward thiol and amine groups enables macrocyclization under mild aqueous conditions without the need for protecting groups.^22^^,^^24^ Under the tested conditions, OPA chemistry generated stable isoindole-linked products and supported analyzable NT-loop and K-loop topologies. Rather than positioning this approach as a replacement for existing cyclic peptide library systems, we view it as a complementary route distinguished by direct access to native proteolytic sequence space together with a rule-based annotation framework for OPA-cyclized products.

A key analytical challenge in cyclic peptide discovery lies in tandem mass spectrometric interpretation,^36^^,^^37^ because ring closure can produce noncanonical fragmentation behavior that is not readily captured by conventional database-search settings. To address this point, we established a fragmentation-rule framework for OPA-cyclized peptides and incorporated it into an automated annotation workflow. This strategy enabled large-scale identification of cyclic peptides from complex libraries and provides a basis for future refinement of cyclic peptide spectral interpretation. While recent advances in computational and AI-driven peptide design have expanded the toolkit for cyclic peptide discovery,^5^ many such approaches remain linked to the availability, accuracy, or interpretability of target structural information. In this context, the present chemistry-enabled, proteome-informed workflow provides a complementary empirical route for identifying protein-binding peptide candidates directly from native proteolytic sequence space.

As a proof of concept, we applied this platform to affinity selection against human IgG, a therapeutically and diagnostically important target.^38^^,^^39^ Affinity capture coupled with LC-MS/MS identified multiple cyclic peptide binder candidates, among which IgG-P1-loop was selected for downstream evaluation. The available binding-related evidence, including SPR-based measurements, HDX-MS, molecular docking, serum pull-down, and imaging-associated labeling experiments, supports IgG-P1-loop as a cyclization-dependent IgG-binding candidate under the present workflow. The apparent SPR-determined *K*_D_ values should be interpreted as supportive estimates under the tested assay format, because independent replicate measurements were not performed. The observed cross-species binding trend is also consistent with recognition of a relatively conserved region within the IgG constant domain, although the strength of this conclusion should be interpreted within the scope of the current data.

Beyond this specific example, the workflow described here offers several practical features. It leverages native peptide diversity, requires limited synthetic input at the discovery stage, and can be integrated with established proteomics pipelines.^40^^,^^41^ Because the library is derived directly from proteolytic digests, its accessible sequence space can in principle be expanded by incorporating proteomes from different biological sources, tissues, or species. In addition, compatibility with comparative proteomic analysis may make this approach useful for future screening efforts across distinct biological states.

Consistent with the bifunctional reactivity of OPA, intermolecular crosslinking products were observed alongside intramolecular macrocycles. Although these species were not the main focus of the present analysis, they may represent an additional and chemically distinct interaction space for future investigation. The current workflow is also restricted to cysteine-containing peptides, which narrows the accessible sequence space^42^; incorporation of orthogonal cyclization chemistries could further broaden the range of peptide substrates that can be explored. While the present rule-based annotation strategy proved effective under the current workflow, integration of data-driven models may further improve annotation performance and scalability. More broadly, future development of this platform will require continued optimization of reaction conditions, analytical workflows, and validation strategies for different application settings.

In summary, we present a proteome-informed, OPA-based workflow for cyclic peptide library construction and annotation at proteomic scale. By linking chemical macrocyclization with LC-MS/MS-readable topology assignment, this approach expands the available toolkit for cyclic peptide discovery and provides a complementary route for identifying protein-binding peptide candidates from native proteolytic sequence space.

### Limitations of the study

This study has several limitations. The current workflow relies on cysteine-containing peptides and therefore samples a defined subset of proteolytic sequence space. OPA-mediated condensation can also generate intermolecular products, whereas downstream analyses in this study focused primarily on confidently identified intramolecular macrocycles. For the IgG proof of concept, given the low signal intensity and rapid association and dissociation, the SPR-derived affinity values should be interpreted as apparent estimates under the tested assay format, and target selectivity cannot be concluded from SPR data alone. Finally, HDX-MS and docking provide a plausible structural model for the observed interaction, although future studies will be required to define the binding interface at residue-level resolution.

## Resource availability

### Lead contact

Further information and requests for resources should be directed to and will be fulfilled by the lead contact, Haiteng Deng (dht@tsinghua.edu.cn).

### Materials availability

This study did not generate unique new reagents.

### Data and code availability

The mass spectrometry proteomics data generated in this study have been deposited to the ProteomeXchange Consortium via the PRIDE partner repository^43^ with the dataset identifier PRIDE: PXD074548. This paper does not report original code. All other data supporting the findings of this study are available within the article and its supplemental information files. Any additional information required to reanalyze the data reported in this paper is available from the lead contact upon reasonable request.

## Acknowledgments

This research was supported by the following funding sources: the 10.13039/501100001809National Natural Science Foundation of China (grant nos. T2293763 and 82172556), the 10.13039/501100012166National Key Research and Development Program of China (grant no. 2021YFA1302601), the Beijing Natural Science Foundation (grant no. IS24040), and the Open Research Fund of the State Key Laboratory of Complex, Severe, and Rare Diseases (no. 2025-I-PY-001). Special acknowledgment is given to the Facility for Protein Chemistry and Proteomics at Tsinghua University for their expert technical assistance in sample analysis.

## Author contributions

Y.W., H.D., and W.Z. conceived and designed the study. Y.W. performed the majority of the experiments, including OPA chemistry development, library construction, and bio-affinity screening. K.F., W.H., and Y.X. assisted with sample preparation. X.T. provided technical assistance with HDX-MS data processing. H.D. supervised the project and provided strategic guidance. Y.W. drafted the manuscript, with critical revisions and intellectual input from H.D., W.Z., and all other authors. All authors reviewed and approved the final version of the manuscript.

## Declaration of interests

Y.W., H.D., and W.Z. are listed as inventors on a Chinese patent application related to the OPA-mediated cyclic peptide library construction, identification, screening, and applications described in this manuscript, filed by Tsinghua University and Yangtze Delta Region Institute of Tsinghua University, Jiaxing, Zhejiang (application no. 202610653124.2). The other authors declare no competing interests.

## Declaration of generative AI and AI-assisted technologies in the writing process

During the preparation of this manuscript, the authors used ChatGPT (OpenAI) for language polishing and improving clarity of expression. After using this tool, the authors carefully reviewed and edited the content and take full responsibility for the scientific accuracy and integrity of the work.

## STAR★Methods

### Key resources table

**Table:** 

REAGENT or RESOURCE	SOURCE	IDENTIFIER
**Antibodies**		

EGF Receptor Antibody	Cell Signaling Technology	Cat# 2232; RRID: AB_331707
PCNA (D3H8P) Rabbit Monoclonal Antibody	Cell Signaling Technology	Cat# 13110; RRID: AB_2636979

**Biological samples**		

Normal human serum	Sigma-Aldrich	Cat# S1-M

**Chemicals, peptides, and recombinant proteins**		

Human IgG	ACMEC	Cat# AC63311
Rabbit IgG	ACMEC	Cat# AC63318
Mouse IgG	ACMEC	Cat# AC63315
Rat IgG	ACMEC	Cat# AC63316
Bovine serum albumin (BSA)	Sigma-Aldrich	Cat# A9418
Sequencing Grade Modified Trypsin	Promega	Cat# V5111
*Ortho*-phthalaldehyde (OPA)	Sigma-Aldrich	Cat# P1378; CAS: 643–79–8
Dithiothreitol (DTT)	Solarbio	Cat# D8220; CAS: 3483–12–3
Iodoacetamide (IAA)	Sigma-Aldrich	Cat# I1149; CAS: 144–48–9
2-Mercaptoethylamine	Sigma-Aldrich	Cat# 641022
2,2′-Dithiodipyridine (DTDP)	Sigma-Aldrich	Cat# D5767; CAS: 2127–03–9
Deuterium oxide (D2O)	J&K	Cat# 261750; CAS: 7789–20–0
5-FITC-maleimide	MCE	Cat# HY-W998680; CAS: 2228857–33–6
IgG-P1-Line	Bankpeptide Biological Technology Co., Ltd.	Custom synthesized
FcIII	Bankpeptide Biological Technology Co., Ltd.	Custom synthesized

**Critical commercial assays**		

BCA Protein Assay Kit	Beyotime	Cat# P0012

**Deposited data**		

Mass spectrometry proteomics data	This paper; PRIDE ProteomeXchange	PRIDE: PXD074548
Human IgG1 Fc domain structure	Protein DataBank	PDB: 5W5L
UniProt human database	UniProt	N/A

**Experimental models: Cell lines**		

Human: HEK293T cells	ATCC	CRL-3216
Human: immortalized human nasal epithelial cells (HNEpCs)	WHELAB	Cat# C1291

**Software and algorithms**		

Proteome Discoverer v2.3	Thermo Fisher Scientific	v2.3
pLink 3	Institute of Computing Technology, CAS	http://pfind.ict.ac.cn/software/pLink/
Biacore Evaluation Software	Cytiva	N/A
DockThor portal	DockThor	https://dockthor.lncc.br/
Protein-Ligand Interaction Profiler (PLIP)	PLIP	https://plip-tool.biotec.tu-dresden.de/
R v3.6.2	R Project	https://www.r-project.org/
tidyverse	R package	https://www.tidyverse.org/
ggplot2	R package	https://ggplot2.tidyverse.org/
ggpubr	R package	https://rpkgs.datanovia.com/ggpubr/
ComplexHeatmap	R package	https://bioconductor.org/packages/ComplexHeatmap/
clusterProfiler	R package	https://bioconductor.org/packages/clusterProfiler/

**Other**		

NHS-Activated Sepharose 4 Fast Flow	Cytiva	Cat# 17090601
Series S Sensor Chip CM5	Cytiva	Cat# 29104988
Sep-Pak C18 cartridges	Waters	Cat# WAT054945
Orbitrap Fusion Lumos Tribrid mass spectrometer	Thermo Fisher Scientific	Instrument
EASY-nLC 1200 system	Thermo Fisher Scientific	Instrument
Biacore T200 SPR system	Cytiva	Instrument
Biacore 8 K+ SPR system	Cytiva	Instrument
Zeiss Axio Observer inverted microscope	Zeiss	Instrument

### Experimental model and study participant details

#### Cell lines and culture conditions

HEK293T cells (ATCC, CRL-3216) and immortalized human nasal epithelial cells (HNEpCs; WHELAB, Cat. No. C1291) were used in this study. HEK293T cells were cultured in DMEM supplemented with 10% fetal bovine serum and 1% penicillin-streptomycin. HNEpCs were cultured in epithelial cell medium according to the supplier’s instructions. Cells were maintained at 37°C in a humidified incubator with 5% CO_2_ and were confirmed to be mycoplasma-free.

### Method details

#### Proteome preparation and enzymatic digestion

HEK293T cells cultured in 15-cm dishes were washed twice with PBS (Wisent, Canada) and lysed in a buffer containing 1% (w/v) SDS (Solarbio, China) and 1% (v/v) PMSF (Beyotime, China) in PBS. Cells were collected by scraping, denatured at 95 °C for 5 min, and further disrupted using a cell homogenizer combined with ultrasonication (30% power, 6 cycles of 5 s with 10 s intervals) on ice. The lysate was centrifuged at 14,000 × g for 15 min at 4 °C to remove cell debris, and the supernatant was collected. Total proteins were precipitated with four volumes of pre-cooled acetone at −20 °C overnight. The protein pellets were resuspended in 8 M urea buffered with PBS (pH 7.4), and protein concentrations were determined using a BCA protein assay kit (Beyotime).

For proteome digestion, protein samples were reduced with 10 mM dithiothreitol (DTT, Solarbio) at 55 °C for 30 min. To prepare the control group for pre-enrichment analysis, cysteine residues were alkylated with 50 mM iodoacetamide (IAA, Sigma-Aldrich) in the dark for 30 min. For samples destined for subsequent thiol enrichment and OPA-mediated cyclization, the alkylation step was omitted. The urea concentration was then diluted to 1 M with PBS (pH 7.4), and proteins were digested with sequencing-grade trypsin (Promega, USA) at an enzyme-to-substrate ratio of 1:50 (w/w) at 37 °C for 16 h. The reaction was quenched with 1% (v/v) trifluoroacetic acid (TFA, Thermo Fisher). Peptides were desalted using Sep-Pak C18 cartridges (Waters) according to the manufacturer’s instructions. Briefly, the cartridges were washed with 0.1% (v/v) TFA, and peptides were eluted with 80% (v/v) acetonitrile containing 0.1% TFA. The eluates were concentrated to dryness using a vacuum centrifugal concentrator (G-Bio, China).

#### Selective enrichment of cysteine-containing peptides

Thiol-reactive resins were prepared and activated for the covalent capture of cysteine-containing peptides. Briefly, NHS-activated Sepharose 4 FF resin (Cytiva) was functionalized with 2-mercaptoethylamine (Sigma-Aldrich) to generate thiol groups. The resin was then activated with 500 mM 2,2′-dithiodipyridine (DTDP, Sigma-Aldrich) in 50% (v/v) acetonitrile (ACN) at room temperature for 4 h in the dark. The activated resin was washed sequentially with 1% (v/v) acetic acid in 50% ACN and PBS (pH 7.4) to remove excess DTDP and adjust to neutral pH.

For peptide enrichment, dried tryptic peptides reconstituted in PBS (pH 7.4) were incubated with the activated resin (1 mL wet volume) at room temperature for 2 h with gentle rotation. To reduce nonspecific background, non-specifically bound peptides were removed by sequential washing (5 mL each, twice) with PBS, 1% (v/v) TFA, and 1% (v/v) TFA in 50% ACN. Resin-bound cysteine-containing peptides were released by incubation with 50 mM DTT in PBS at room temperature for 1 h, followed by two additional washes with 50 mM DTT in 50% ACN containing 1% TFA. The combined eluates were concentrated to approximately 200 μL using a vacuum centrifugal concentrator (G-Bio, China), desalted via Sep-Pak C18 cartridges (Waters), and evaporated to dryness. The resulting cysteine-peptide library with free sulfhydryl groups was stored at −20 °C for subsequent OPA-mediated cyclization.

#### OPA-mediated cyclization and LC-MS/MS analysis

The enriched cysteine-containing peptides were cyclized using *ortho*-phthalaldehyde (OPA, Sigma-Aldrich) under mild aqueous conditions. Dried peptide pellets were reconstituted in 50 mM PBS (pH 7.4) and reacted with 0.15 mM OPA for 30 min at room temperature in the dark. The reaction was quenched by addition of 10 mM Tris buffer to consume excess OPA. This process facilitated intramolecular condensation between the cysteine thiol and either the N-terminal α-amino or lysine ε-amino group, yielding a thioisoindole-linked cyclic structure. The resulting products were desalted, concentrated, and resuspended in 0.1% (v/v) formic acid (Thermo Scientific) for LC-MS/MS analysis.

Peptides were analyzed using an Orbitrap Fusion Lumos Tribrid mass spectrometer coupled with an EASY-nLC 1200 system (Thermo Scientific). Peptides were separated on a C18 column (75 μm × 25 cm, 2 μm) using a 155-min gradient (5%–95% acetonitrile) at a flow rate of 300 nL/min. Mass spectra were acquired in data-dependent acquisition (DDA) mode, with the top 20 precursors selected for HCD fragmentation at 30% normalized collision energy.

#### Affinity screening against human IgG

To identify candidate binders, the OPA-cyclized peptide library was screened against human IgG. Human IgG (ACMEC, China) was immobilized onto NHS-activated Sepharose 4 FF resin (Cytiva). The resin was washed with cold PBS (pH 7.4) and incubated with IgG (2 mg/mL) for 2 h at room temperature with gentle rotation. Unreacted active sites were quenched with 1 M ethanolamine (pH 8.0) for 10 min. For affinity selection, the cyclized library was incubated with the IgG-conjugated resin for 2 h. Non-specifically bound peptides were removed by sequential washing with PBS containing 0.05% (v/v) Tween 20 and PBS. Bound peptides were eluted using 10% (v/v) acetic acid for 10 min, and the eluates were concentrated using a vacuum centrifugal concentrator (G-Bio, China) for LC-MS/MS analysis. LC-MS/MS instrumental parameters and chromatographic gradients were identical to those described above.

#### Cyclic peptide identification and annotation

To automate the identification of OPA-bridged cyclic peptides, a computational workflow was implemented based on specific mass modeling and fragmentation rules. Database searching was performed using Proteome Discoverer (v2.3, Thermo Scientific) or pLink 3 (Institute of Computing Technology, CAS), with oxidation of methionine and acetylation of the protein N-terminus set as default variable modifications.

To model OPA-mediated macrocyclization, the condensation of OPA with amines and thiols was defined as a ring-opening event during HCD fragmentation. In this model, the N-terminus or Lys side-chain amine linked with OPA was assigned a mass shift of +130.9950 Da (△m = m(OPA) − m(4H) − m(2O) + m(S) + m(H)), while the Cys site was assigned a mass shift of −32.9793 Da (△m = −m(S) − m(H)), corresponding to formation of dehydroalanine (Dha). This configuration accounts for the characteristic total mass increase of +98.0157 Da (△m = m(OPA) − m(4H) − m(2O)) resulting from OPA bridge formation.

#### Surface plasmon resonance (SPR) analysis

The linear precursor peptide IgG-P1-Line and FcIII were synthesized via Fmoc solid-phase peptide synthesis (Bankpeptide Biological Technology Co., Ltd., Hefei, China) and purified by HPLC. IgG-P1-Line was subjected to OPA-mediated cyclization in PBS buffer (pH 7.4) to generate IgG-P1-loop for binding analysis. The interactions of IgG-P1-loop, IgG-P1-Line, and FcIII with IgG were evaluated by SPR using a Biacore T200/8 K+ SPR system (Cytiva). IgG species, including human IgG, rabbit IgG, rat IgG, and mouse IgG (ACMEC), were immobilized onto a Series S Sensor Chip CM5 (Cytiva, Cat. No. 29104988) via standard amine coupling in 10 mM sodium acetate buffer (pH 4.0–5.0) to achieve a target immobilization level. The running buffer consisted of PBS (pH 7.4) containing 0.05% (v/v) Tween 20. Peptide analytes at concentrations ranging from 0 to 160 nM were injected at a flow rate of 30 μL/min for 90 s, followed by 90 s dissociation. SPR data were analyzed using Biacore Evaluation software, and binding values are reported as apparent SPR-determined dissociation constants (*K*_D_ values). SPR measurements were performed as single concentration-series experiments; therefore, replicate-based SD or SEM values were not calculated.

#### Hydrogen–deuterium exchange mass spectrometry (HDX–MS)

To map the binding interface of the IgG–peptide complex, HDX–MS was performed. Human IgG was pre-incubated with selected cyclic peptides (or vehicle control), then exposed to deuterium oxide (D_2_O) buffer. After designated time points (0 s, 30 s, 90 s, 300 s, and 600 s), deuterium incorporation was quenched with ice-cold buffer (pH 2.5), and samples were digested using pepsin resin (Thermo Scientific). The resulting peptides were analyzed by LC-MS/MS. Changes in deuterium uptake (ΔHDX) were mapped onto the IgG structure to identify protected or altered regions associated with peptide binding.

#### Molecular docking simulations

Molecular docking was performed using the DockThor portal^44^^,^^45^ and Protein–Ligand Interaction Profiler (PLIP) tools. The crystal structure of the human IgG1 Fc domain (PDB: 5W5L) was used as the docking receptor. The search space was defined to encompass the CH2–CH3 interface based on the HDX–MS results. Docking was executed using the standard genetic algorithm with default parameters. Predicted binding poses were analyzed for hydrogen bonding, hydrophobic contacts, and interaction hotspots.

#### Serum pulldown assay

Cyclic peptide IgG-P1-loop and its linear control (IgG-P1-Line) were conjugated to maleimide- or NHS-activated resins in PBS (0.5 mM, 2 h, room temperature). After quenching with cysteamine or ethanolamine, resins were washed and incubated with 100-fold diluted normal human serum (Sigma-Aldrich) at 4 °C for 2 h. Unbound proteins were removed by alternating PBS and PBS-Tween washes. Bound IgG was eluted with 10% acetic acid, neutralized with Tris, and analyzed by SDS-PAGE and Coomassie staining. IgG enrichment was quantified by densitometry of the ∼55 kDa heavy-chain band, normalized to human serum albumin (HSA), and compared between loop and linear peptide groups.

#### Fluorescence labeling with FITC-conjugated peptides

For labeling assays, cells were fixed with 4% paraformaldehyde (PFA, Sigma-Aldrich) for 15 min and washed with PBS. Permeabilization was performed with 0.2% Triton X-100 (Sigma-Aldrich) when labeling nuclear antigens. After blocking with 5% bovine serum albumin (BSA, Sigma), cells were incubated with anti-PCNA or anti-EGFR antibody (Cell Signaling Technology) at 1:200 dilution. After washing, FITC-labeled cyclic peptide probe (10 μL, ∼5 μg/mL) was added and incubated for 1 h at room temperature in the dark. Nuclei were counterstained with DAPI (1 μg/mL, Thermo Scientific), and fluorescence images were acquired using a Zeiss Axio Observer inverted microscope.

### Quantification and statistical analysis

#### Mass spectrometry data processing

All LC-MS/MS raw data acquired in DDA mode were processed using Proteome Discoverer (v2.3) or pLink 3. Database searching was performed using the Sequest HT algorithm against the UniProt human database or a custom cyclic peptide library. Peptide-spectrum matches (PSMs) and protein identifications were filtered to a false discovery rate (FDR) of <5%. To identify OPA-cyclized peptides, pLink 3 was applied using high-precision spectral matching, and the results were filtered at a 5% FDR threshold. Outputs (including pep.xml and quantification tables) were used for downstream statistical evaluation.

#### Statistical analysis

Bioinformatics and statistical analyses were conducted in R (v3.6.2). Data preprocessing was performed using the tidyverse package, and visualizations were generated using ggplot2 and ggpubr. Hierarchical clustering was implemented with ComplexHeatmap, while Gene Ontology (GO) enrichment analysis was conducted using clusterProfiler. Statistical comparisons between groups were performed using two-tailed Student’s t-tests or one-way ANOVA followed by Tukey’s post hoc test. Significance levels were defined as follows: *p* < 0.05 (∗), *p* < 0.01 (∗∗), *p* < 0.001 (∗∗∗), and *p* < 0.0001 (∗∗∗∗); “ns” indicates non-significance (*p* > 0.05).

## Supplemental information

Document S1. Figures S1–S9

Table S1. Peptides identified before and after thiol-affinity enrichment

Table S2. OPA-cyclized peptides identified from BSA-derived peptide libraries

Table S3. OPA-cyclized peptides and related products identified from the HEK293T proteome-derived peptide library
