# Supplementary material for: Development of proteome-derived cyclic peptide libraries via ortho-phthalaldehyde-assisted cyclization
Source: iScience. 2026 Sep 22;29(10):117598. doi: 10.1016/j.isci.2026.117598 (PMC13632188; doi:10.1016/j.isci.2026.117598)
Supplement: Supplementary file 1 [file mmc1.pdf]

iScience, Volume *29*

Supplemental information

Development of proteome-derived cyclic peptide libraries via ortho-phthalaldehyde-assisted cyclization

Ying Wei, Kangze Feng, Wenxuan Hou, Xiaolin Tian, Yuanyuan Xue, Wenhao Zhang, and Haiteng Deng

Supplemental figures and legends

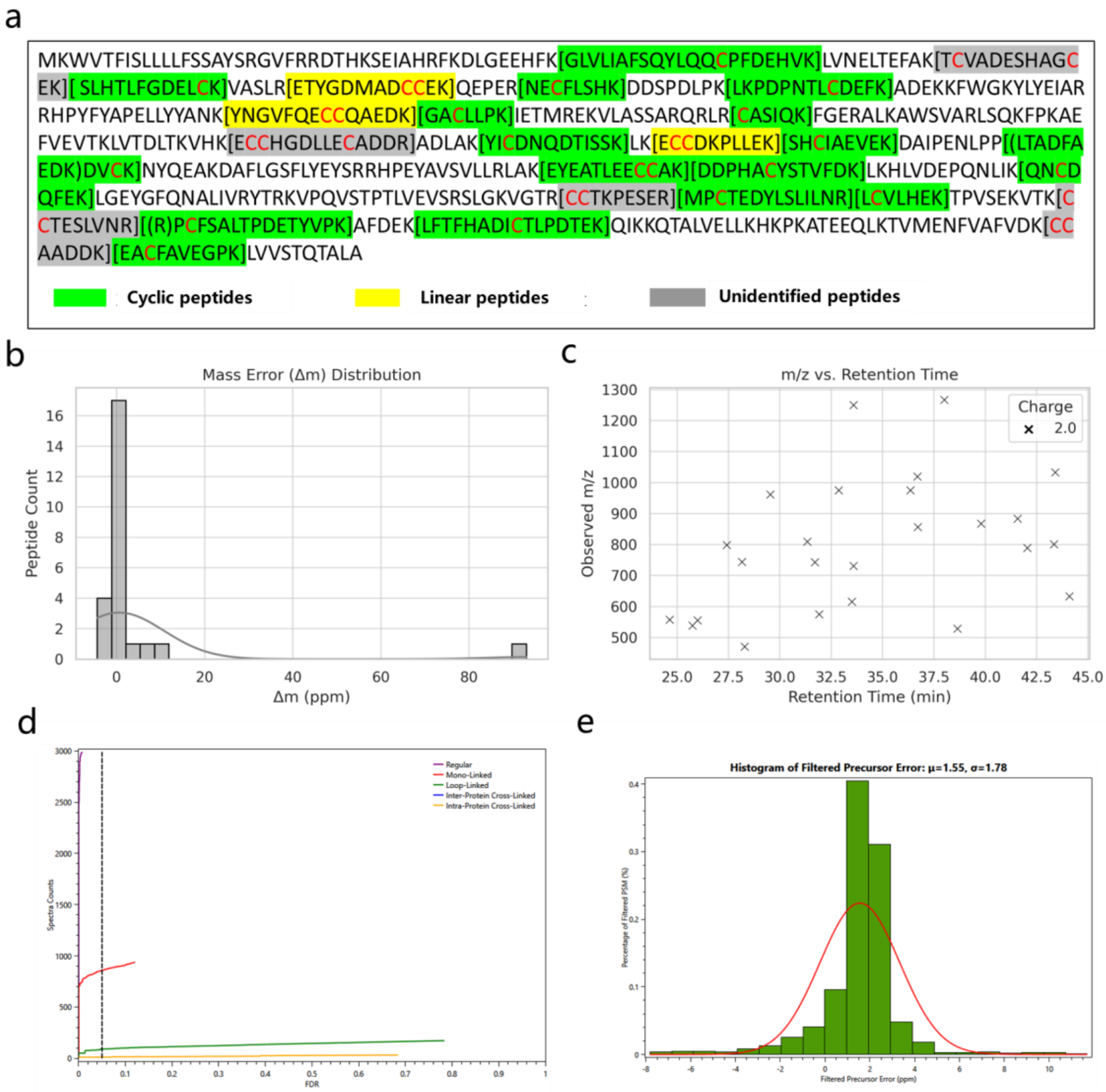

Figure S-1. Quality control and validation of the OPA-cyclized BSA model peptide library.
(a) Coverage map of the full-length bovine serum albumin (BSA) sequence after tryptic digestion. Peptides containing cysteine residues are highlighted, with cysteine (C) residues marked in red. Peptides detected with OPA-induced cyclized conformations are shown in green, peptides detected only in unreacted (linear) form are shown in yellow, and peptides not detected in either form are shown in grey. (b) Mass error distribution (Δm, ppm) of peptides identified as cyclized species (NT-loop or K-loop) by Proteome Discoverer 2.3 ($q < 0.05$), showing that the majority of precursor mass deviations are within ±5 ppm and follow a near-Gaussian distribution. (c) Two-dimensional scatter plot of precursor m/z versus chromatographic retention time for identified cyclized peptides, demonstrating uniform distribution across the LC gradient and m/z range, consistent with diverse physicochemical properties and reliable chromatographic behavior. (d) Distribution of peptide spectrum matches (PSMs) for different linkage types as a function of false discovery rate (FDR) in pLink 3 search results, showing that a substantial number of loop-linked (cyclic) peptides are retained below the 5% FDR threshold. (e) Histogram of filtered precursor mass errors for pLink 3-identified cyclized peptides after FDR control, exhibiting a Gaussian-like distribution with a mean of 1.55 ppm and a standard deviation of 1.78 ppm.

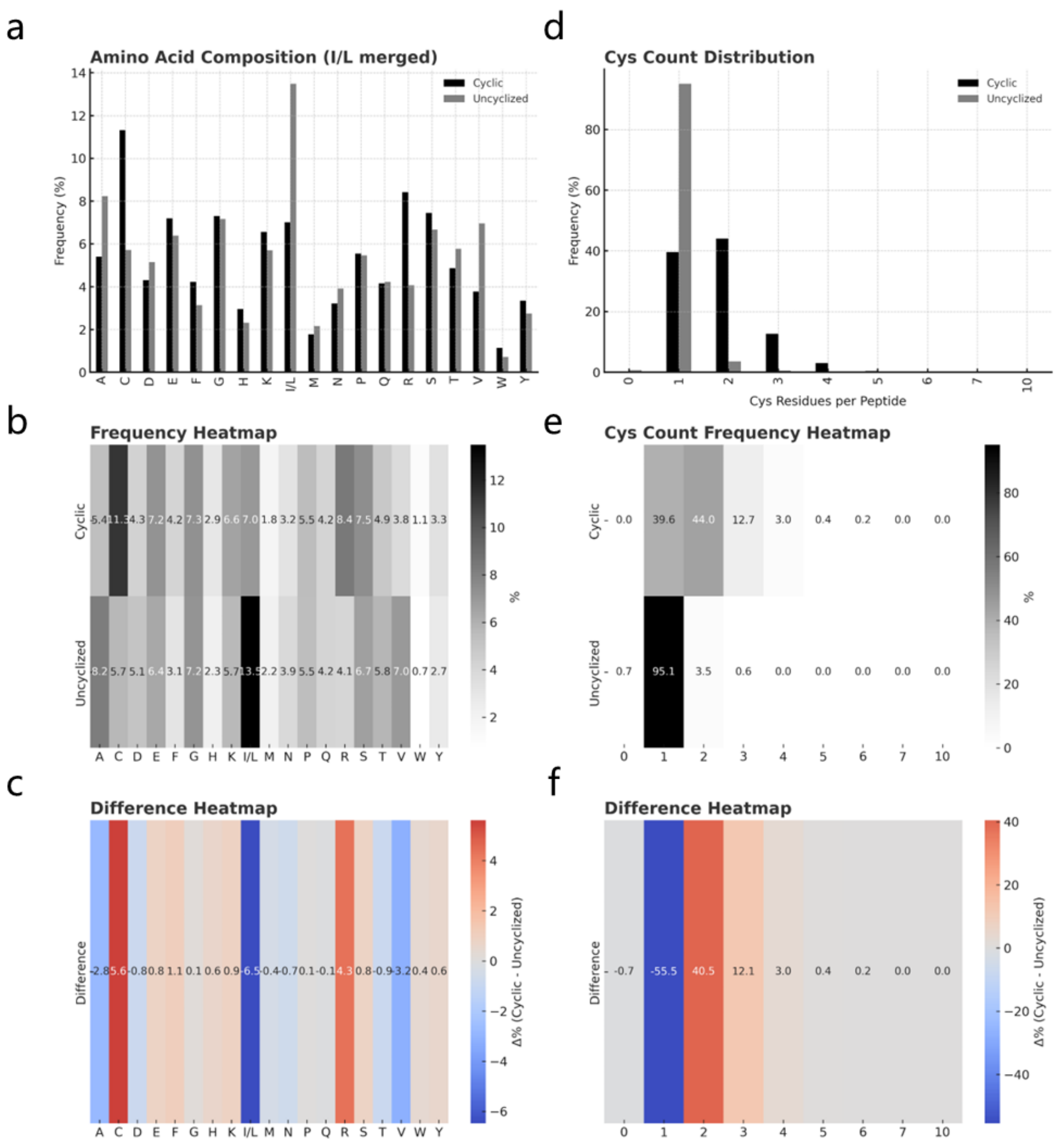

Figure S-2. Amino Acid Composition Analysis of Uncyclized and Cyclic Peptides.

This figure compares the amino acid frequency distribution and Cys residue count between cyclic peptides and their uncyclized counterparts before undergoing OPA reaction. (a-c) Amino acid composition comparison: (a) Bar chart illustrating the amino acid composition distribution of both groups, with the Cyclic group showing higher proportions of reactive residues such as C, W, and H compared to the Uncyclized group. (b) Heatmap displaying amino acid frequencies, where color intensity indicates the relative abundance of each amino acid in both groups. (c) Differential heatmap (Cyclic - Uncyclized), with red indicating higher proportions in the Cyclic group and blue in the Uncyclized group, highlighting the strongest enrichment of Cys, which correlates with its increased density in the cyclic peptides. (d-f) Comparison of Cys residue distributions: (d) Bar chart showing the proportion of peptides containing varying numbers of Cys residues, with the Cyclic group demonstrating a higher proportion of peptides with two or more Cys residues. (e) Frequency heatmap illustrating the distribution of Cys residues in both groups. (f) Differential heatmap, with red indicating a higher proportion of Cys residues in the Cyclic group and blue in the Uncyclized group. The enrichment of multi-Cys peptides in the cyclization reaction likely

results from the spatial closure advantages provided by multiple reactive sites, as well as the natural enrichment of multi-Cys fragments in native protein structures.

This dual analysis of amino acid composition and Cys residue distribution indicates preferential recovery of Cys-enriched peptides under the tested OPA cyclization workflow.

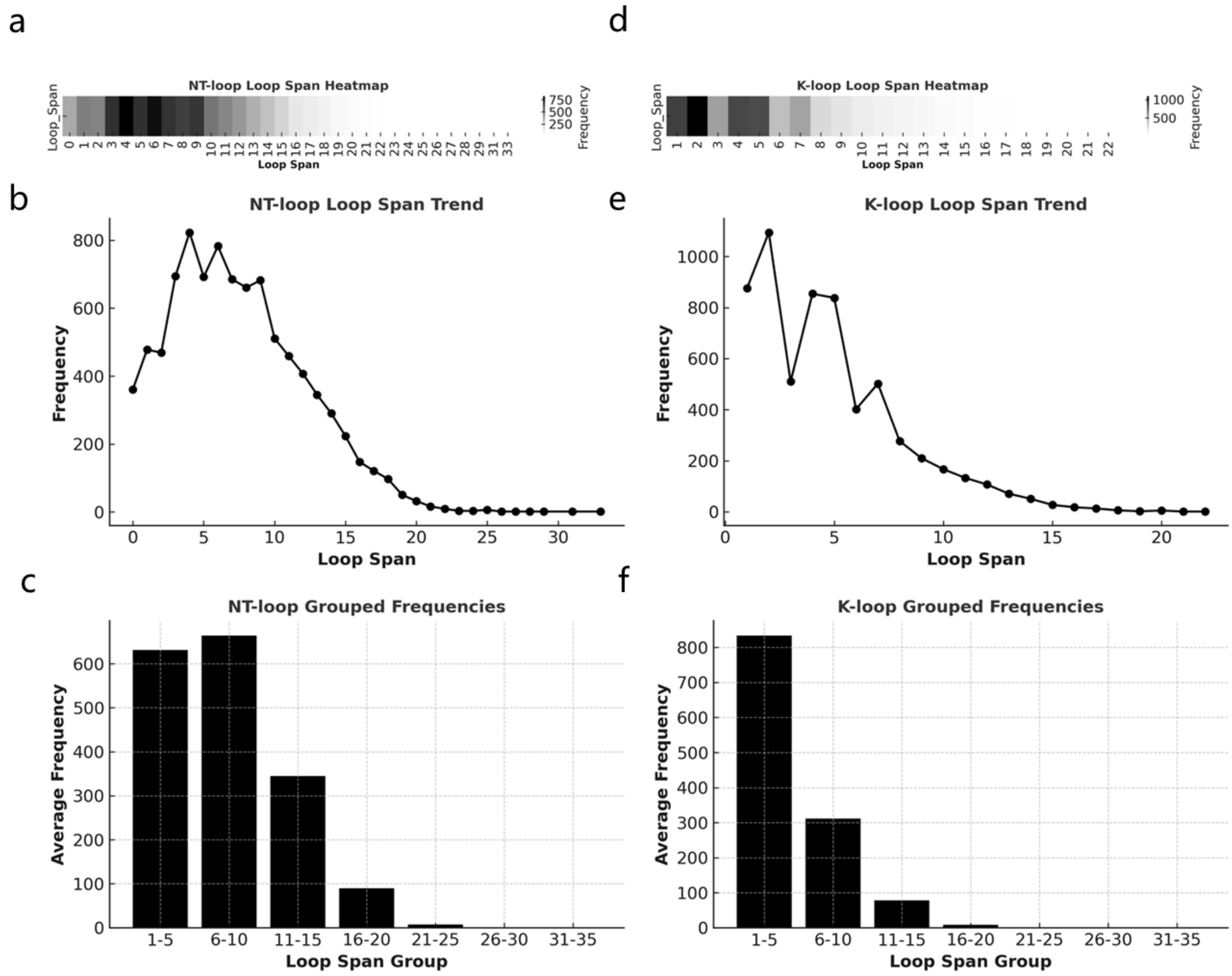

Figure S-3. Loop Span Distribution Analysis of Cyclic Peptides with NT-loop and K-loop Configurations. This figure shows the loop span distribution of cyclic peptides with NT-loop and K-loop configurations. (a) Heatmap of loop span frequencies for NT-loop peptides, with an enrichment in the 3-7 aa range. (b) Frequency curve for NT-loop peptides, peaking at 5-7 aa and gradually decreasing, with a small proportion in the 10-15 aa range. (c) Grouped bar chart for NT-loop peptides, showing a dominant frequency in the short spans (1-5 and 6-10 aa). (d) Heatmap of loop span frequencies for K-loop peptides, with higher frequencies in shorter spans (1-3 aa). (e) Frequency curve for K-loop peptides, showing a sharp decline as the span increases, with the highest frequency in the 1-3 aa range. (f) Grouped bar chart for K-loop peptides, confirming a preference for short loop spans. Both NT-loop and K-loop configurations show a preference for short loop spans, with K-loop having a stronger preference for ultra-short spans. NT-loop peptides, however, can accommodate slightly longer spans (6-10 aa and 11-15 aa), suggesting greater flexibility in loop closure. This preference may reflect the compatibility of compact loop structures with OPA-mediated closure and subsequent mass spectrometry detection.

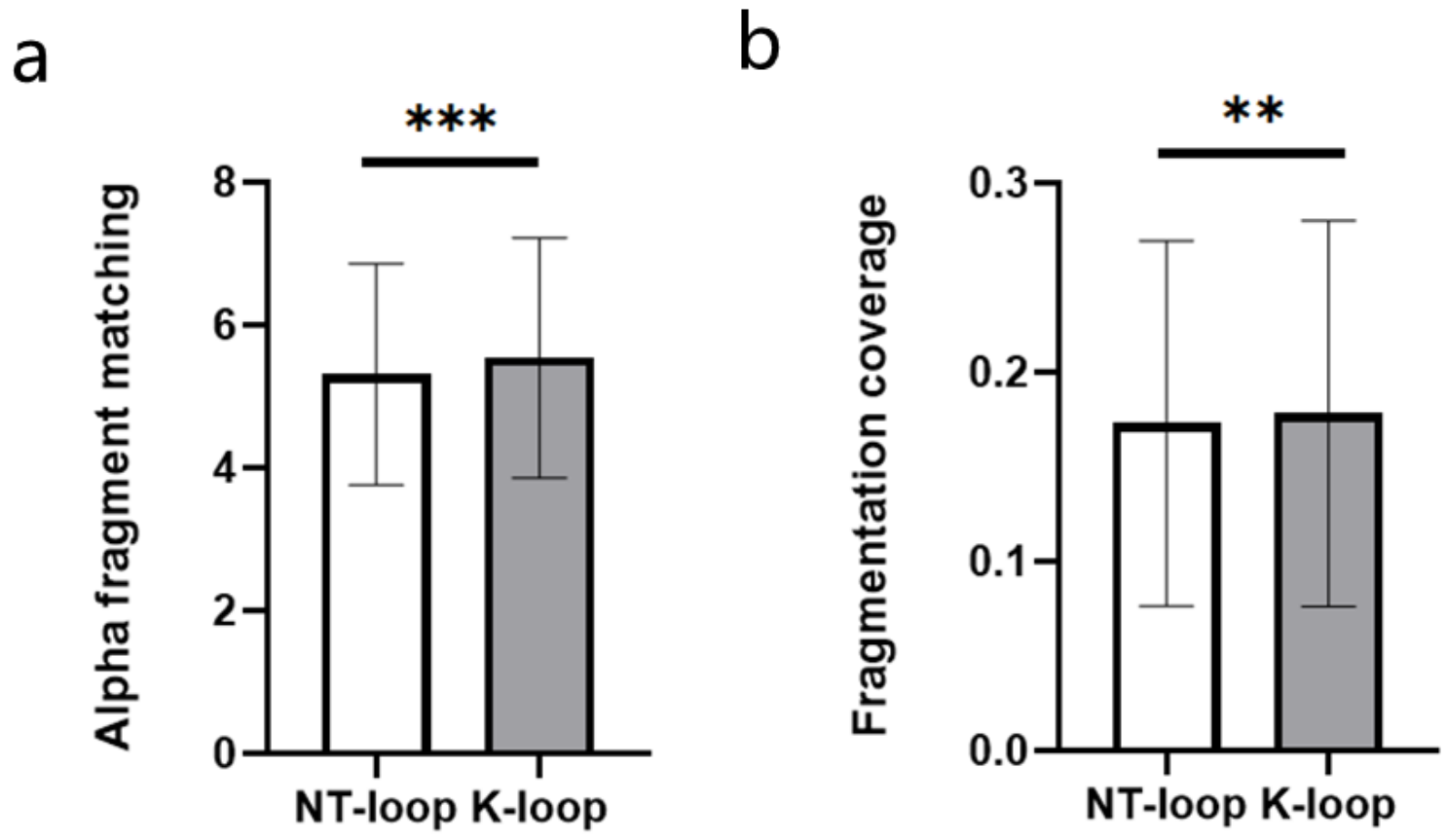

Figure S-4. Alpha Fragment Matching and Fragmentation Coverage of Cyclic Peptides with Different Configurations. This figure presents the fragmentation characteristics of cyclic peptides with two different configurations, based on the constructed natural cyclic peptide database and MS/MS analysis. (a) Alpha Fragment Matching: The number of theoretical Alpha fragmentation ions matched to the experimental spectrum. (b) Fragmentation Coverage: The percentage of the peptide sequence covered by the matched Alpha fragmentation ions. Significance was tested using a two-tailed Welch t-test, with significance levels marked as: $***p < 0.001$, $**p < 0.01$.

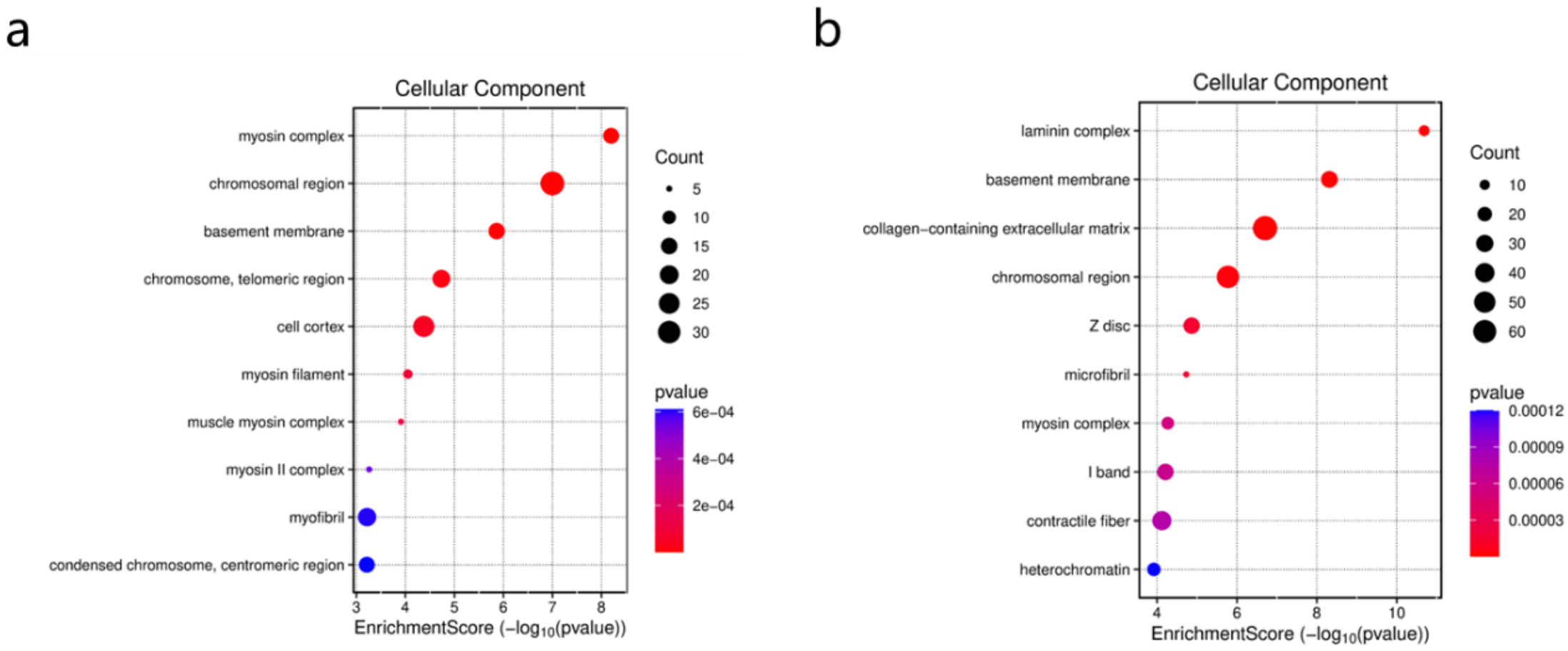

Figure S-5. Cellular Component Enrichment Analysis (GO-CC) of Peptide Source Proteins Before and After OPA-Mediated Cyclization. This figure shows the Gene Ontology (GO) cellular component (CC) enrichment analysis of peptides before and after OPA-mediated cyclization. (a) Before cyclization, significant enrichment is found in cytoskeleton and contraction-related structures such as myosin complex and myofibril, indicating that linear peptides are mainly derived from cytoskeletal proteins. (b) After cyclization, enrichment shifts to extracellular matrix and membrane-associated structures, such as basement membrane and collagen-containing extracellular matrix, indicating enrichment of peptides derived from extracellular proteins in the recovered cyclic peptide set. The size of the points represents the number of proteins, and color indicates enrichment significance (p-value). The x-axis shows the enrichment score (−log10(p-value)).

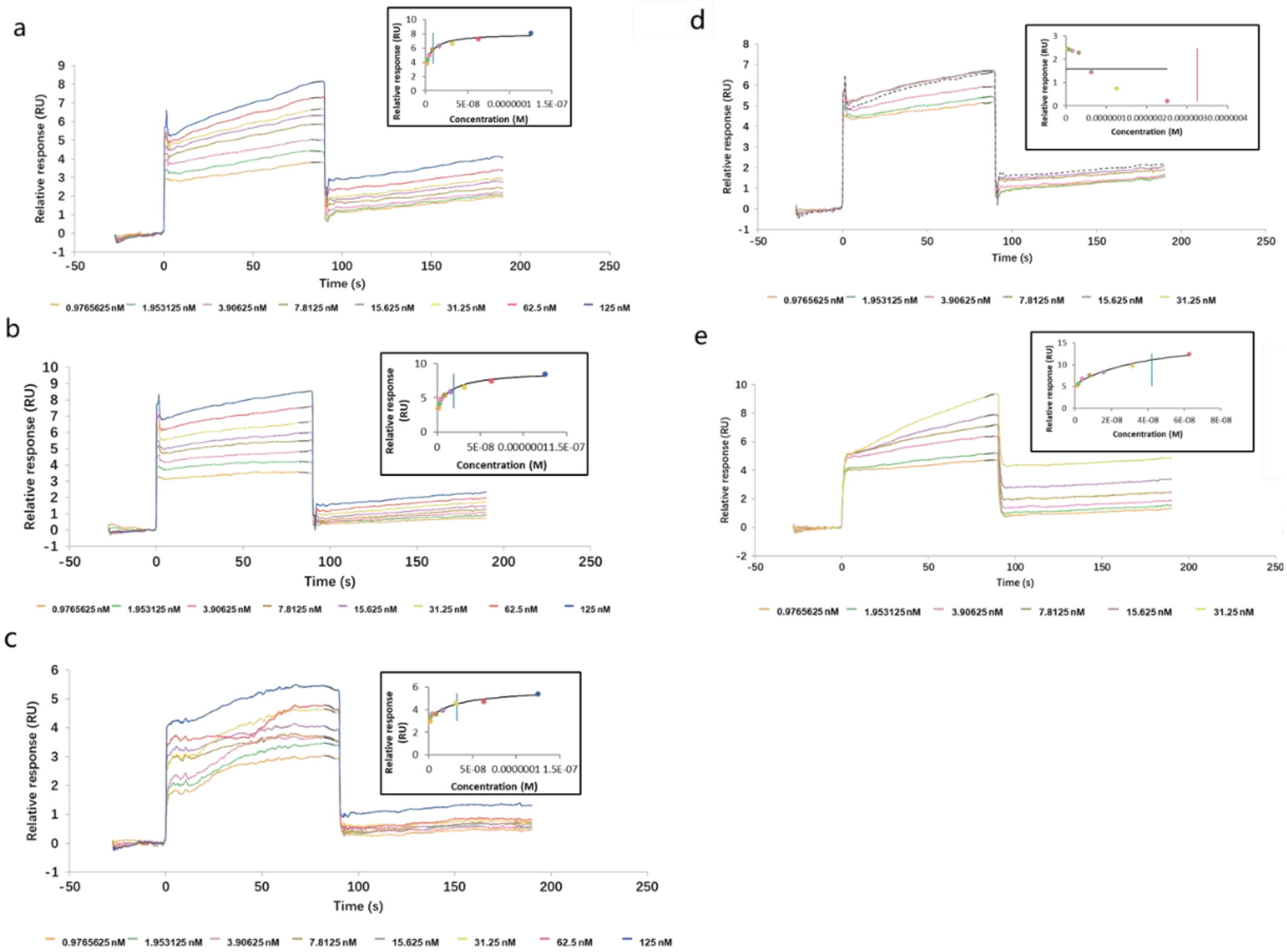

Figure S-6. SPR sensorgrams of IgG-P1-loop measured against rabbit, rat, and mouse IgG, and comparison with IgG-P1-Line and FcIII measured against human IgG. Colored curves represent the experimental sensorgrams, and black solid lines indicate the corresponding steady-state affinity fitting curves where applicable. (a) Surface plasmon resonance (SPR) sensorgrams of IgG-P1-loop measured against immobilized rabbit IgG at the indicated peptide concentrations. The sensorgrams showed concentration-dependent responses. Steady-state affinity fitting yielded an apparent SPR-determined dissociation constant (K_D) of 3.52 nM. (b) SPR sensorgrams of IgG-P1-loop measured against immobilized rat IgG at the indicated peptide concentrations. The sensorgrams showed concentration-dependent responses. Steady-state affinity fitting yielded an apparent SPR-determined dissociation constant (K_D) of 4.62 nM. (c) SPR sensorgrams of IgG-P1-loop measured against immobilized mouse IgG at the indicated peptide concentrations. The sensorgrams showed concentration-dependent responses. Steady-state affinity fitting yielded an apparent SPR-determined dissociation constant (K_D) of 11.0 nM. (d) SPR sensorgrams of IgG-P1-Line measured against immobilized human IgG at the indicated peptide concentrations. The observed responses were substantially weaker than those of IgG-P1-loop under the same assay format, and the concentration-dependent data did not support reliable steady-state affinity fitting. (e) SPR sensorgrams of the positive-control peptide FcIII measured against immobilized human IgG at the indicated peptide concentrations. Steady-state affinity fitting yielded an apparent SPR-determined dissociation constant (K_D) of 8.81 nM, with an R_{max} value of 6.4 RU.

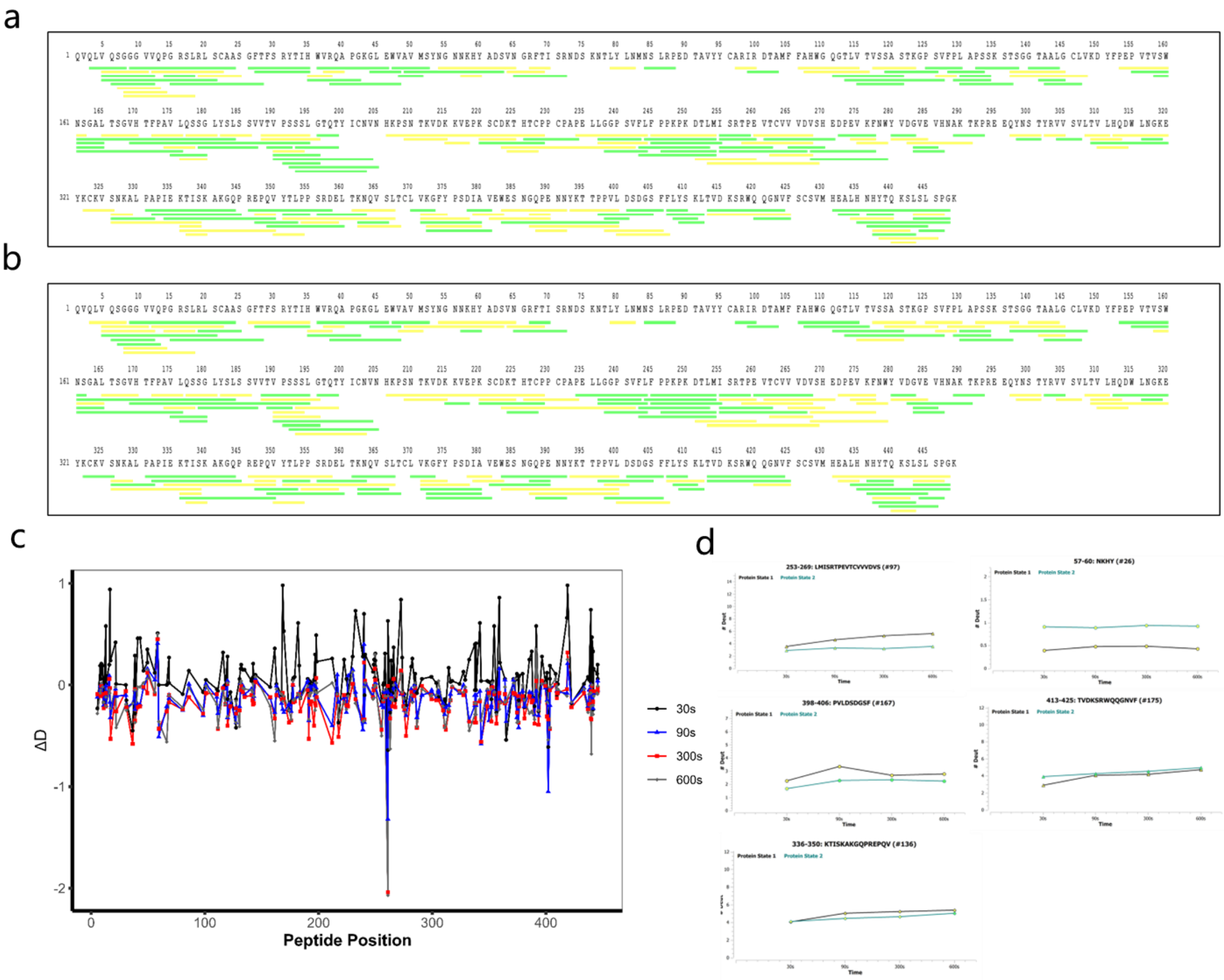

Figure S-7. HDX-MS Analysis of Human IgG Binding to IgG-P1-loop: Peptide Coverage, ΔHDX Distribution, and Exchange Kinetics. (a) Control group (untreated human IgG): The green regions represent high-confidence peptides (⩾95% identification), yellow represents medium-confidence peptides (80-95% identification), and blank areas indicate non-covered regions. Both groups show high-confidence peptide coverage across the majority of the human IgG heavy chain sequence, with an overall coverage rate of 94%. The peptides are widely distributed across the Fc domain, providing a reliable structural foundation for identifying potential binding sites and subsequent ΔHDX comparisons. (b) IgG-P1-loop treatment group: Similar coverage as the control, but showing potential conformational changes in specific regions. (c) ΔHDX distribution curves for different time points: The x-axis represents the starting residue number of the human IgG heavy chain, and the y-axis shows the difference in deuterium exchange (ΔHDX) between the treatment group and control group at various time points (30s, 90s, 300s, 600s). Negative values indicate reduced deuterium exchange in the treatment group, suggesting more stable local conformations or binding shielding; positive values indicate increased exchange, possibly reflecting relaxed conformations or enhanced accessibility. (d) Deuterium exchange kinetics of the five peptides with the most significant ΔHDX changes: The x-axis represents the deuterium exchange reaction time (seconds), and the y-axis shows the average number of deuterium atoms incorporated into the respective peptides. The black curves represent the control group (Protein State 1), and the blue-green curves represent the treatment group (IgG-P1-loop complex, Protein State 2). The five peptides show significant changes in ΔHDX: the peptides LMISRTPEVTCVVVDS (253-269), PVLDSDGSF (398-406), and KTISKAKGQPREPQV (336-350) show decreased deuterium

exchange (negative ΔHDX), suggesting increased conformational stability or binding shielding, potentially corresponding to the cyclic peptide binding interface. The peptides TVDKSRWQQGNVF (413-425) and NKHY (57-60) show increased deuterium exchange (positive ΔHDX), indicating more flexible conformations after binding, likely located at the structural edges or away from the core binding region.

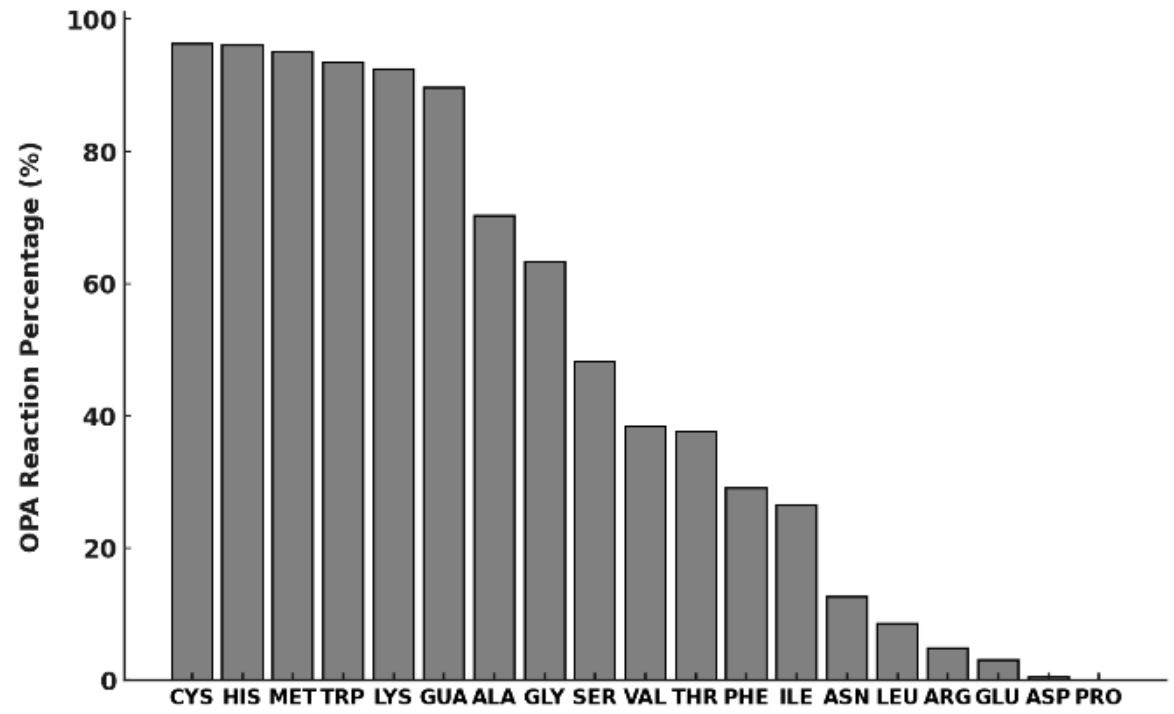

Figure S-8. Reaction rates of 20 amino acids with OPA in PBS at pH 7.4. This figure illustrates the apparent reaction rates of 20 natural L-amino acids with ortho-phthalaldehyde (OPA) in PBS at pH 7.4 over 10 min. The results show that cysteine (Cys) reacts most rapidly and completely with OPA, depleting almost all of the OPA in the solution within 10 minutes (residual OPA < 5%), and is set as the reference value of 100%. Other amino acids, such as lysine (Lys) and histidine (His), which contain potential nucleophilic groups (amine, imidazole), also show some reactivity, but lower than Cys. Amino acids with acidic side chains, such as glutamic acid (Glu) and aspartic acid (Asp), exhibit very low reactivity with OPA, with almost no change in the residual OPA. These observations are consistent with higher apparent OPA reactivity for thiol-containing Cys and lower apparent reactivity for acidic amino acids under the tested conditions.

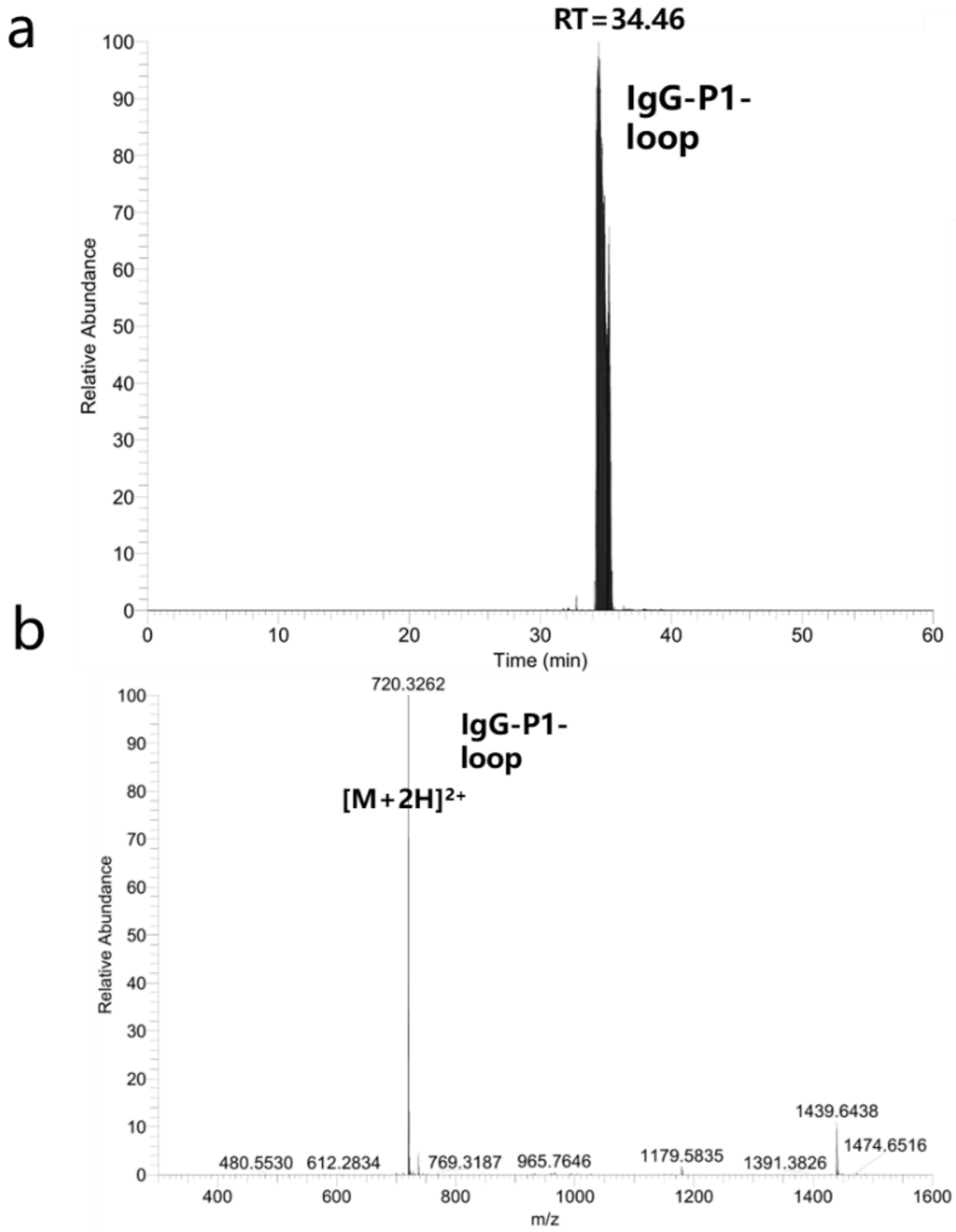

Figure S-9. LC–MS characterization of the cyclic peptide IgG-P1-loop. (a) Reverse-phase HPLC chromatogram of IgG-P1-loop showing a predominant peak at approximately 34 min, indicating high purity of the cyclized product. (b) Corresponding electrospray ionization mass spectrum (ESI-MS) displaying a major ion at m/z 720.3262, assigned to the doubly protonated species $[M+2H]^{2+}$. The observed mass is consistent with the calculated molecular weight of IgG-P1-loop, supporting formation of the cyclized product. No prominent signals corresponding to unreacted linear peptide or detectable side products were observed under the tested conditions, consistent with the purity of the product.
